# Algal symbiosis adaptively restructures the cnidarian microbiome to enhance holobiont metabolism

**DOI:** 10.1038/s41467-026-79238-6

**Published:** 2026-11-02

**Authors:** Guoxin Cui, David K. Ngugi, Migle Konciute, Si Cheng, Kit Xi Liew, Jianing Mi, Salim Al-Babili, Manuel Aranda

**Affiliations:** 1 Simon F.S. Li Marine Science Laboratory, School of Life Sciences, The Chinese University of Hong Kong, Hong Kong SAR, China; 2 King Abdullah University of Science and Technology (KAUST), Biological and Environmental Science and Engineering Division, Red Sea Research Platform, Thuwal, Saudi Arabia; 3 Division of Marine Ecology, GEOMAR Helmholtz Centre for Ocean Research Kiel, Research Unit Marine Symbioses, Kiel, Germany; 4 State Key Laboratory of Traditional Chinese Medicine Syndrome, The Second Affiliated Hospital of Guangzhou University of Chinese Medicine, Guangzhou, China; 5 King Abdullah University of Science and Technology (KAUST), Biological and Environmental Science and Engineering Division, The BioActives Lab, Thuwal, Saudi Arabia; 6 King Abdullah University of Science and Technology (KAUST), Biological and Environmental Science and Engineering Division, The Plant Science Program, Thuwal, Saudi Arabia

**Keywords:** Marine biology, Molecular ecology, Metabolomics, Symbiosis

## Abstract

Microbiomes are integral to the function and resilience of cnidarian holobionts, yet the spatial and functional integration of microbial communities within host tissues remains poorly understood. Here, we show that symbiosis with photosynthetic dinoflagellates induces adaptive, tissue-specific restructuring of the microbiome in the cnidarian model Aiptasia, fundamentally enhancing holobiont nitrogen assimilation and metabolic flexibility. Using laser microdissection, tissue-resolved metatranscriptomics, gnotobiotic hosts, and stable isotope tracing, we reveal that symbiosis profoundly restructures microbial communities across the epidermis and gastrodermis, enriching for taxa and pathways that support nitrogen assimilation in photosynthetically active tissues. Expression of microbial nitrite reductase is strongly upregulated in the symbiotic gastrodermis, and ^15^N-tracer experiments demonstrate that bacterial assimilation of nitrite synergizes with algal symbionts to fuel the biosynthesis of essential amino acids. These findings show that symbiosis drives spatially compartmentalized microbiome adaptation within animal tissues, aligning microbial metabolism with host-algal nutrient exchanges to enhance holobiont functioning.

## Abstract (web summary)

Cnidarian-algal symbioses help corals thrive in nutrient-poor oceans. This study shows that algal symbiosis reshapes tissue-specific microbiomes, promoting bacterial nitrogen assimilation and essential amino acid production.

## Introduction

Cnidarian-algae symbioses form the foundation of coral reef ecosystems, among the most biodiverse and productive ecosystems on Earth despite their nutrient-poor environments^1,2^. These partnerships between cnidarians and photosynthetic dinoflagellates of the family Symbiodiniaceae enable efficient nutrient cycling and energy transfer, underlying the metabolic success of the holobiont in oligotrophic environments^3^. While extensive research has illuminated the physiological and ecological aspects of these symbioses, much of the focus has been on the animal-algae relationship, leaving the broader principles of microbiome functional integration underexplored^4^.

Advances in metabarcoding-based metagenomics have revealed the holobiont nature of cnidarians, which include not only the host animal and Symbiodiniaceae but also diverse microorganisms such as bacteria, archaea, fungi, and viruses^5–8^. Microbial communities are integral to cnidarian holobionts, implicated in key processes such as nitrogen and carbon cycling, pathogen defense, modulation of host immunity, and the biosynthesis of vitamins and amino acids. The microbial partners inhabit distinct compartments within the cnidarian host, such as the surface mucus layer^9–11^, gastrovascular cavity^12,13^, skeleton^14^, and tissues^15,16^. Each of these compartments represents a unique physicochemical microhabitat with potentially distinct microbial communities and functional roles. However, tissues, which constitute a major component of these compartments, are often treated as a uniform microenvironment, overlooking how their structural and functional compartmentalization might support holobiont function. Imaging-based studies suggest potential tissue-specific bacterial distributions^17–19^, but comprehensive investigations at the functional level are lacking. In particular, it remains unclear how symbiosis with Symbiodiniaceae reshapes the microbial communities and their functional roles across tissue layers and symbiotic states, or how these changes contribute to holobiont metabolism and function.

While metagenomic analyses have provided valuable insights into cnidarian-associated bacterial communities, these approaches typically link bacterial presence to inferred functions^20,21^. These associations often rely on assumptions and lack experimental validation, leaving unresolved questions about how bacteria actively support or influence the holobiont.

Here, we use the cnidarian model Aiptasia to dissect how symbiosis restructures the spatial distribution and functional roles of microbes to enhance holobiont functioning. We hypothesize that the bacterial community is highly compartmentalized between tissue layers and dynamically regulated by the presence of the algal symbiont. To test this, we first characterize the spatial composition of the tissue-specific microbiome using 16S rRNA gene sequencing. Next, to distinguish between baseline microbial presence and active metabolic output, we perform metatranscriptomic profiling to identify symbiosis-induced shifts in gene expression and functional pathways. Finally, to assess how microbiome restructuring influences host nutrient acquisition, we employ stable isotope probing (^15^N) coupled with targeted amino acid profiling. By comparing gnotobiotic (bacteria-depleted) and regular anemones across symbiotic states, we separate the metabolic contributions of bacteria from those of the algal symbiont. Through this integrated approach, we show that symbiosis reshapes microbial composition and activity across tissue layers, significantly enhancing holobiont metabolic capacity. In particular, synergistic nitrogen assimilation and the production of essential amino acids emerge as key processes linking microbial activity, tissue specificity, and symbiotic states.

## Results

### Taxonomic classification of active tissue-specific microbes

To examine how symbiosis influences microbial communities at the functional and tissue-specific level, we generated deep transcriptomic data (42.6 ± 1.4 million reads; mean ± s.e.m.) from the gastrodermis (Gas) and epidermis (Epi) of aposymbiotic (Apo) and symbiotic (Sym) Aiptasia (*n* = 4 biological replicates; 16 samples total; Supplementary Data 1)^3^. By employing a targeted sequencing approach using a combination of random hexamers and a poly-T chimeric primer, we comprehensively captured mRNAs and rRNAs from host, algal symbionts, and associated microbes. This approach enabled us to resolve the tissue-specific functional interplay among these partners.

**Fig. 1:**
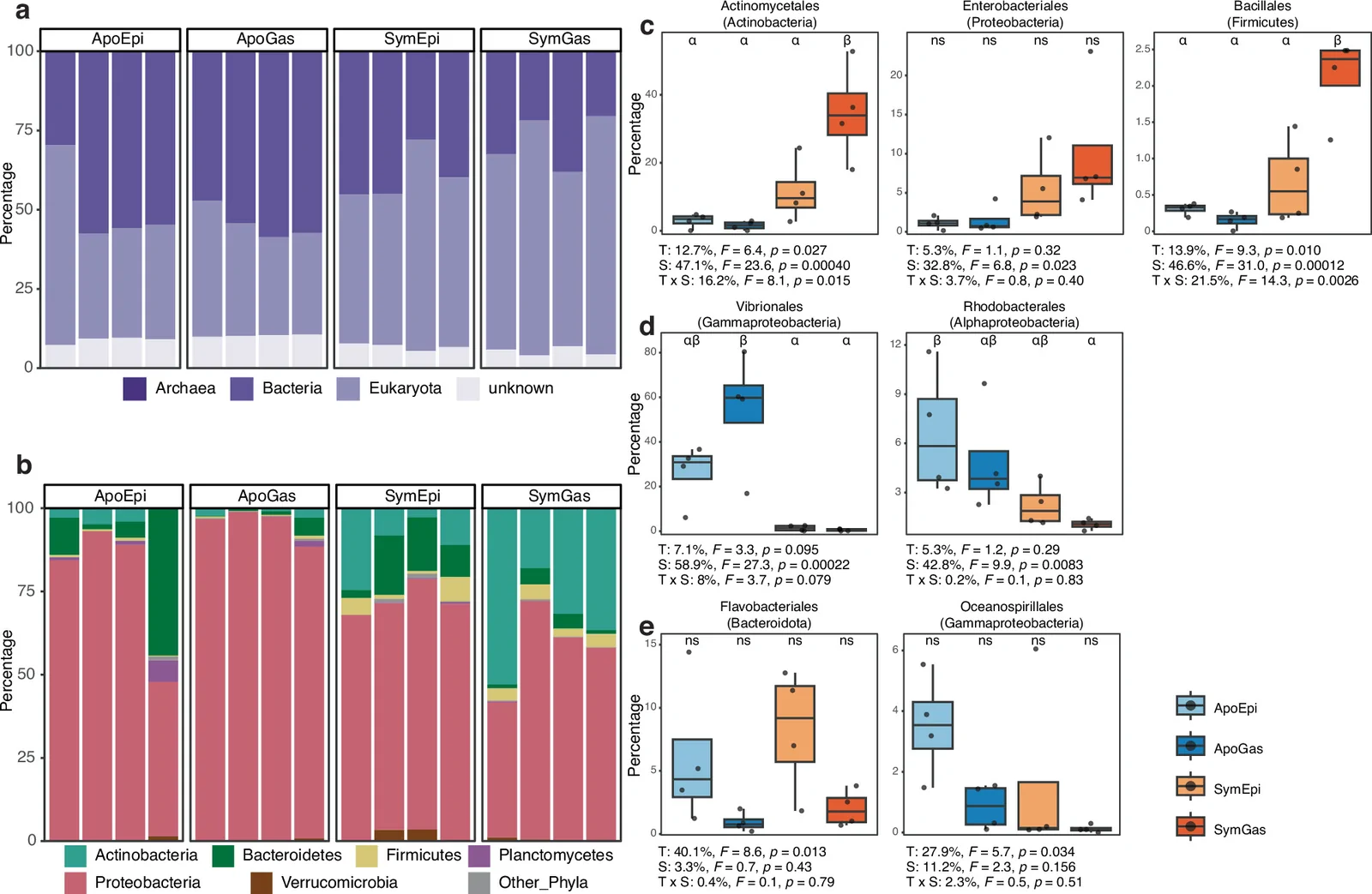
Symbiosis-induced tissue-specific bacterial community composition. **a** Relative ribosomal RNA abundance at the domain level across four tissue types. Archaea constitute <1% of the relative abundance across all samples. Apo, aposymbiotic; Sym, symbiotic; Epi, epidermis; Gas, gastrodermis. **b** Bacterial community composition at the phylum level, providing an overview of taxonomic profiles across the four tissue types. Finer taxonomic resolutions for the microbial communities (family and genus levels) are provided in Supplementary Data 2. Differentially abundant bacterial taxa at the order level, categorized as enriched in response to symbiosis (**c**), suppressed by symbiosis (**d**), or specific to the epidermal tissue layer (**e**). For all boxplots, center lines represent the median, bounds of boxes indicate the interquartile range (IQR), whiskers extend to the minimum and maximum values within 1.5 × IQR, and individual data points represent biological replicates (*n* = 4 biologically independent samples per group). Statistical significance and variance partitioning were assessed using two-way analysis of variance (ANOVA), with different letters above boxes denoting significant differences between groups based on Tukey’s HSD post hoc test (*p* < 0.05). Full statistical results are provided in Supplementary Data 9. Source data are provided as a Source Data file.

From these data, we first isolated rRNA reads and assembled 16S rRNAs, yielding a total of 17.9 million 16S rRNA transcripts across all samples. Taxonomic classification assigned most of these transcripts to eukaryotic (49.3 ± 3.8%) or bacterial (42.9 ± 3.3%) origins (Fig. 1a). Further classification of the bacterial 16S transcripts revealed that the majority belonged to Proteobacteria (75.4 ± 4.5%), followed by Actinobacteria (12.7 ± 4.0%), Bacteroidetes (7.9 ± 2.8%), and Firmicutes (2.1 ± 0.55%) (Fig. 1b).

To resolve community shifts with higher taxonomic resolution, we examined bacterial composition at the order level and found that multiple bacterial orders varied significantly across tissue layers in response to the symbiotic state of the anemones (Fig. 1c–e; full statistical results in Supplementary Data 9). Actinomycetales (Actinobacteria) Enterobacterales (Proteobacteria), and Bacillales (Firmicutes) were significantly enriched in symbiotic anemones (two-way ANOVA, adjusted *p* < 0.05), with Actinomycetales and Bacillales showing specific enrichment in the symbiotic gastrodermis (Fig. 1c, Tukey’s HSD post hoc, *p* < 0.05). In contrast, Vibrionales (Gammaproteobacteria) and Rhodobacterales (Alphaproteobacteria) were significantly enriched in aposymbiotic anemones (two-way ANOVA, adjusted *p* < 0.05), with Vibrionales representing the most abundant bacterial order in both tissue layers from aposymbiotic Aiptasia (Fig. 1d). Additionally, Flavobacteriales (Bacteroidota) and Oceanospirillales (Gammaproteobacteria) were significantly enriched in the epidermis of the sea anemone Aiptasia independent of their symbiotic state (Fig. 1e; two-way ANOVA, adjusted *p* < 0.05).

### Metatranscriptomic profiling of active microbes

**Fig. 2:**
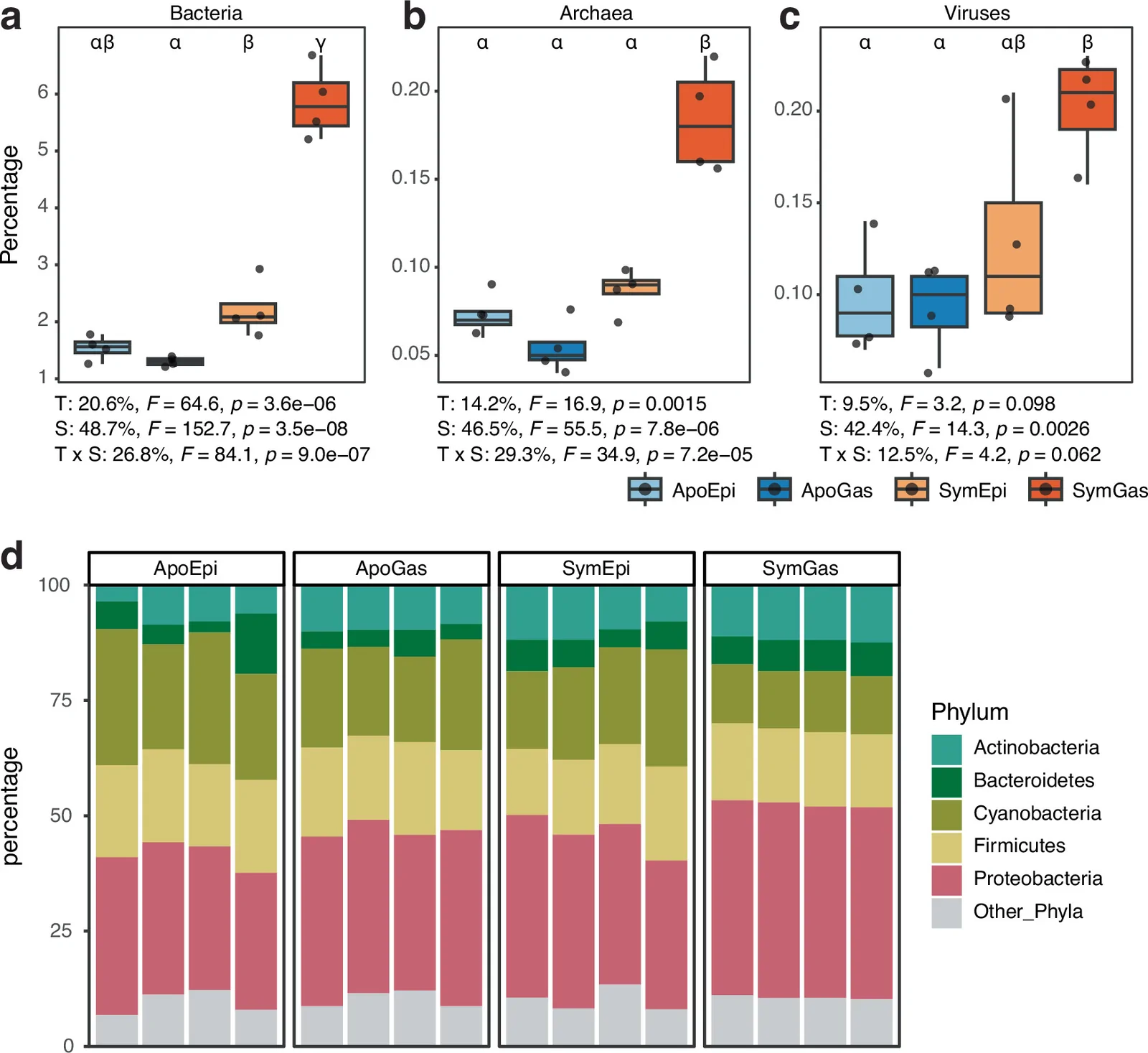
Metatranscriptomic profiles in Aiptasia. Relative abundances of bacteria (**a**), archaea (**b**), and viruses (**c**) across the four tissue types in the sea anemone Aiptasia. **d** mRNA-transcripts-based bacterial community composition at the phylum level across the four tissue types. For panels **a–c**, data are presented as boxplots where center lines represent the median, bounds indicate the interquartile range (IQR), and whiskers extend to the minimum and maximum values within 1.5 × IQR (*n* = 4 biological replicates per group). Statistical significance and variance partitioning were assessed using two-way analysis of variance (ANOVA), with different letters denoting significant differences between groups based on Tukey’s HSD post hoc test (*p* < 0.05). Full statistical results provided in Supplementary Data 10, 11. Source data are provided as a Source Data file.

To further investigate the functional activity and symbiosis-driven changes in the associated microbiome, we assembled mRNA transcripts from the tissue-specific RNA-seq data after filtering out rRNA sequences. We retrieved 1.37 ± 0.06 million microbial reads per sample (mean ± s.e.m.; 3.0 ± 0.5% of total mRNA reads; Fig. 2a–c). These reads were assembled into 10,670 microbial transcripts, comprising coding sequences from bacteria, archaea, and viruses. Notably, the relative abundances of all three microbial groups were significantly increased in symbiotic anemones (Fig. 2a–c; Supplementary Data 10, two-way ANOVA, adjusted *p* < 0.05). However, their spatial distribution differed among groups: bacteria and archaea were specifically enriched in the gastrodermis of symbiotic animals (Fig. 2a, b, Tukey’s HSD post hoc, *p* < 0.05), whereas viral relative abundance was primarily associated with symbiotic state rather than tissue layer alone (Fig. 2c; two-way ANOVA, Symbiosis adjusted *p* = 0.0026, Tissue adjusted *p* = 0.098), with post hoc comparisons indicating elevated levels in the symbiotic gastrodermis relative to aposymbiotic tissues (*p* < 0.05).

Bacterial mRNA transcripts were predominantly classified into five major phyla (Fig. 2d): Proteobacteria (36.6 ± 1.0%; mean ± s.e.m.), Cyanobacteria (20.1 ± 1.3%), Firmicutes (17.9 ± 0.5%), Actinobacteria (9.6 ± 0.6%), and Bacteroidetes (5.7 ± 0.6%). Statistical analysis further showed that the relative abundances of Proteobacteria, Cyanobacteria, and Actinobacteria were significantly influenced by both tissue layer and symbiotic state, whereas Firmicutes varied significantly only with symbiotic state; no significant effects were detected for Bacteroidetes (two-way ANOVA, adjusted *p* < 0.05; Supplementary Data 11). Although the dominant microbial taxa largely overlapped between the rRNA- and mRNA-based profiles, Cyanobacteria were notably absent from the highly abundant taxa in the rRNA pool (Fig. 1b) yet emerged with a high relative abundance among protein-coding transcripts (Fig. 2d), indicating a low abundance but transcriptionally active population with significant spatial variation (Supplementary Data 11). The major contributors to the mRNA pool also did not perfectly mirror the rRNA-based compositional profiles, likely reflecting differences in methodological sensitivity: rRNA-based profiling captures total bacterial abundance, whereas mRNA-based analysis reflects active transcription and is influenced by microbial metabolic states rather than mere physical presence.

To extend taxonomic resolution beyond the phylum-level summaries, we applied complementary higher-resolution profiling of the same samples using MetaPro on the mRNA fraction and Kraken2 on the rRNA fraction (Supplementary Data 2 and 3). The MetaPro-based active community showed significant differences in alpha diversity, with observed genus richness influenced by both tissue layer and symbiotic state and Shannon diversity affected by symbiotic state alone (Supplementary Fig. 2; two-way ANOVA, adjusted *p* < 0.05). Beta diversity of the active community was also significantly structured by tissue layer (R^2^ = 0.10), symbiotic state (R^2^ = 0.38), and their interaction (R^2^ = 0.11; two-way PERMANOVA, all *p* < 0.05). In contrast, the Kraken2-based rRNA community showed no significant differences in alpha diversity, although its beta diversity remained significantly associated with both tissue layer (R^2^ = 0.15) and symbiotic state (R^2^ = 0.30, *p* < 0.05), without a significant interaction term (Supplementary Fig. 2; Supplementary Data 3). Together, these community-level analyses indicate that symbiosis and tissue compartmentalization reshape not only microbial composition but also the structure of the transcriptionally active microbiome, motivating a deeper analysis of microbial gene expression and functional activity.

### Symbiosis-induced microbial activity changes

To obtain a global view of microbial transcriptional variation across all samples, we first performed a principal coordinates analysis (PCoA) on log_2_(TPM + 1)-transformed bacterial transcript abundances across the 16 libraries (Supplementary Fig. 3). This analysis showed that microbial expression profiles were structured primarily by both tissue layer and symbiotic state, consistent with a two-way PERMANOVA that identified significant main effects of both factors (*p* = 0.0001 for each), with a marginal interaction (*p* = 0.051). Group dispersions were homogeneous, indicating that the observed separation reflected shifts in centroid position rather than differences in within-group variability.

**Fig. 3:**
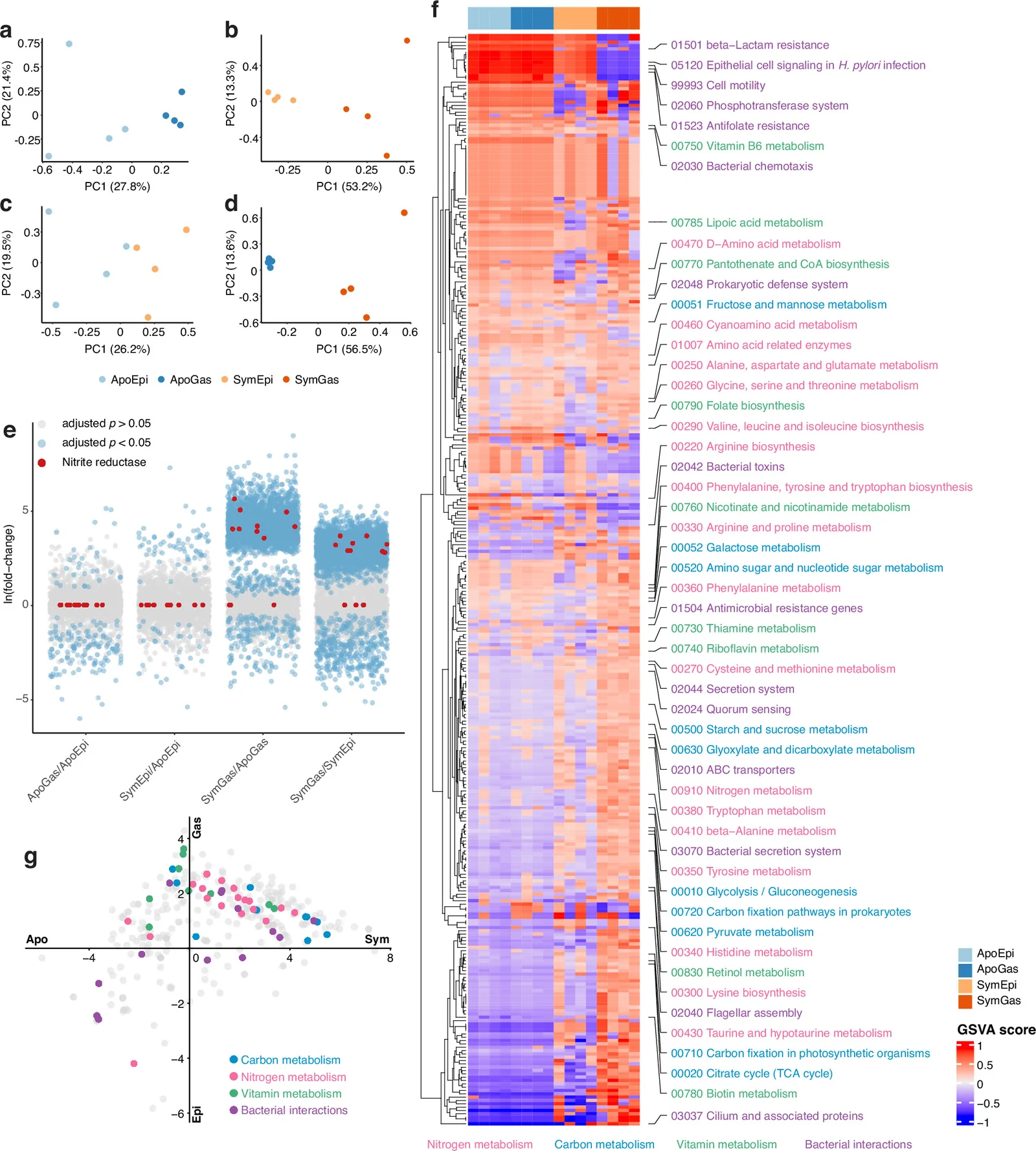
Microbial gene activity changes in response to tissue types and symbiotic states. Principal component analysis (PCA) plots comparing microbial gene expression between ApoGas vs ApoEpi (**a**), SymGas vs SymEpi (**b**), SymEpi vs ApoEpi (**c**), and SymGas vs ApoGas (**d**). **e** Differential expression of microbial transcripts across the four pairwise comparisons. Each point represents a single transcript. Significantly differentially expressed transcripts (*q* < 0.05) are colored blue, non-significant transcripts gray, and nitrite reductase (NiR) transcripts are overlaid in red. **f** Heatmap o**f** gene set variation analysis (GSVA) scores representing pathway activities calculated based on microbial gene expression levels. Pathways are color-coded into four major functional categories. **g** Scatter plot showing differential pathway activity, represented by *t*-values from pairwise comparisons of Sym vs. Apo and Gas vs. Epi. Pathways are color-coded as in (**f**). For all panels, *n* = 4 biological replicates per group. Differential gene expression (**e**) was statistically assessed using the two-sided Wald test via *sleuth*, with p values adjusted for multiple comparisons using Benjamini-Hochberg method. Differential pathway activity (**f**, **g**) was statistically assessed using a two-way factorial linear model with empirical Bayes smoothing via *limma*. All significance thresholds were adjusted for multiple comparisons (adjusted *p* < 0.05). Comprehensive DE results, GSVA scores, and limma outputs are provided in Supplementary Data 5, 7, and 8.

Guided by this global pattern, we next carried out pairwise comparisons between the four tissue types: ApoGas vs. ApoEpi (Fig. 3a), SymGas vs. SymEpi (Fig. 3b), SymEpi vs. ApoEpi (Fig. 3c), and SymGas vs. ApoGas (Fig. 3d). Principal component analyses (PCA) revealed distinct clustering patterns driven by tissue origin and symbiotic state. Crucially, principal component 1 (PC1) explained >50% of the total variance in comparisons involving the symbiotic gastrodermis (SymGas vs. SymEpi, Fig. 3b; SymGas vs. ApoGas, Fig. 3d), compared to only ~27% in the other cmparisons (Fig. 3a, c). This strong separation was mirrored by the differential gene expression results (Supplementary Data 5), with these exact comparisons yielding the largest numbers of differentially expressed genes (DEGs), suggesting that both tissue layer and symbiotic state strongly shape microbial transcriptional responses (Fig. 3e). In particular, the comparison between symbiotic and aposymbiotic gastrodermis was dominated by large-amplitude expression shifts rather than modest regulatory tuning. Most differentially expressed microbial transcripts in this compartment showed large effect sizes, frequently exceeding 2-fold change, consistent with broad transcriptional reprogramming of microbial metabolism driven by the presence of the algal symbiont (Fig. 3e).

To better understand the functional impact of these transcriptional changes, we estimated the activity of metatranscriptome-annotated KEGG pathways across the four tissue types using gene set variation analysis (GSVA; Supplementary Data 7). As visualizing the entirety of the differentially active pathways simultaneously can obscure overarching biological trends, we highlighted a focal subset of the significantly upregulated pathways by manually curating them into four broad thematic categories. These specific categories, namely carbon and nitrogen metabolism, vitamin biosynthesis, and bacterial interaction, were chosen because they capture the foundational metabolic and regulatory dynamics of the symbiosis. Pathways within these categories exhibited significantly higher transcriptional activity in symbiotic anemones, with upregulation occurring either globally across both tissue layers or localized specifically to the gastrodermis (Fig. 3f). To evaluate these functional shifts across the holobiont, we modeled the pathway activity (GSVA scores) using a two-way factorial generalized linear model. This analysis confirmed that most carbon and nitrogen metabolism pathways were significantly driven by the main effect of symbiotic state, with many also exhibiting significant interaction effects that pinpoint their localized upregulation specifically within the symbiotic gastrodermal tissue layer (Fig. 3g; Supplementary Data 8, *limma*, adjusted *p* < 0.05).

Consistent with the enhanced carbon and nitrogen metabolism, gene ontology (GO) enrichment analysis identified nitrate assimilation (GO:0042128) among the most over-represented biological processes, significantly enriched with DEGs from the SymGas vs ApoGas (Fisher’s exact test, *p* = 0.0017) and SymGas vs SymEpi (*p* = 0.0023) comparisons (Supplementary Data 6). Among genes associated with nitrate assimilation, we identified 12 genes encoding nitrite reductases (NiR), of which 9, classified as either *Klebsiella oxytoca* NasB or *Bacillus subtilis* NasD homologs, were significantly upregulated in the symbiotic gastrodermal tissues in the relevant pairwise comparisons (Fig. 3e).

### Bacterial function in essential amino acids synthesis

Cnidarians lack the capacity to synthesize many essential amino acids de novo, a constraint that has been proposed as a key driver for harboring microbial symbionts capable of nutrient provision^22,23^. Because the observed spatial upregulation of *NiR* transcripts physically places active microbial nitrogen metabolism in the tissue layer with the most direct access to photosynthetically fixed carbohydrates, we hypothesized that cnidarian-associated microbes may couple this localized nitrite assimilation with the biosynthesis of essential amino acid. Importantly, the NiR genes most strongly upregulated in the symbiotic gastrodermis originated from the microbial taxa *Klebsiella oxytoca* and *Bacillus subtilis*. These taxa are well-known probiotic symbionts in both plant and animal systems, where they contribute to nitrogen cycling, vitamin production, and essential amino acid biosynthesis^24–27^. To test this hypothesis, we performed ^15^N enrichment analyses of essential amino acids by combining ^15^N-nitrite incubation with ultra-high-performance liquid chromatography-high-resolution mass spectrometry (UHPLC-HR-MS) in both gnotobiotic and control anemones, under symbiotic and aposymbiotic conditions.

**Fig. 4:**
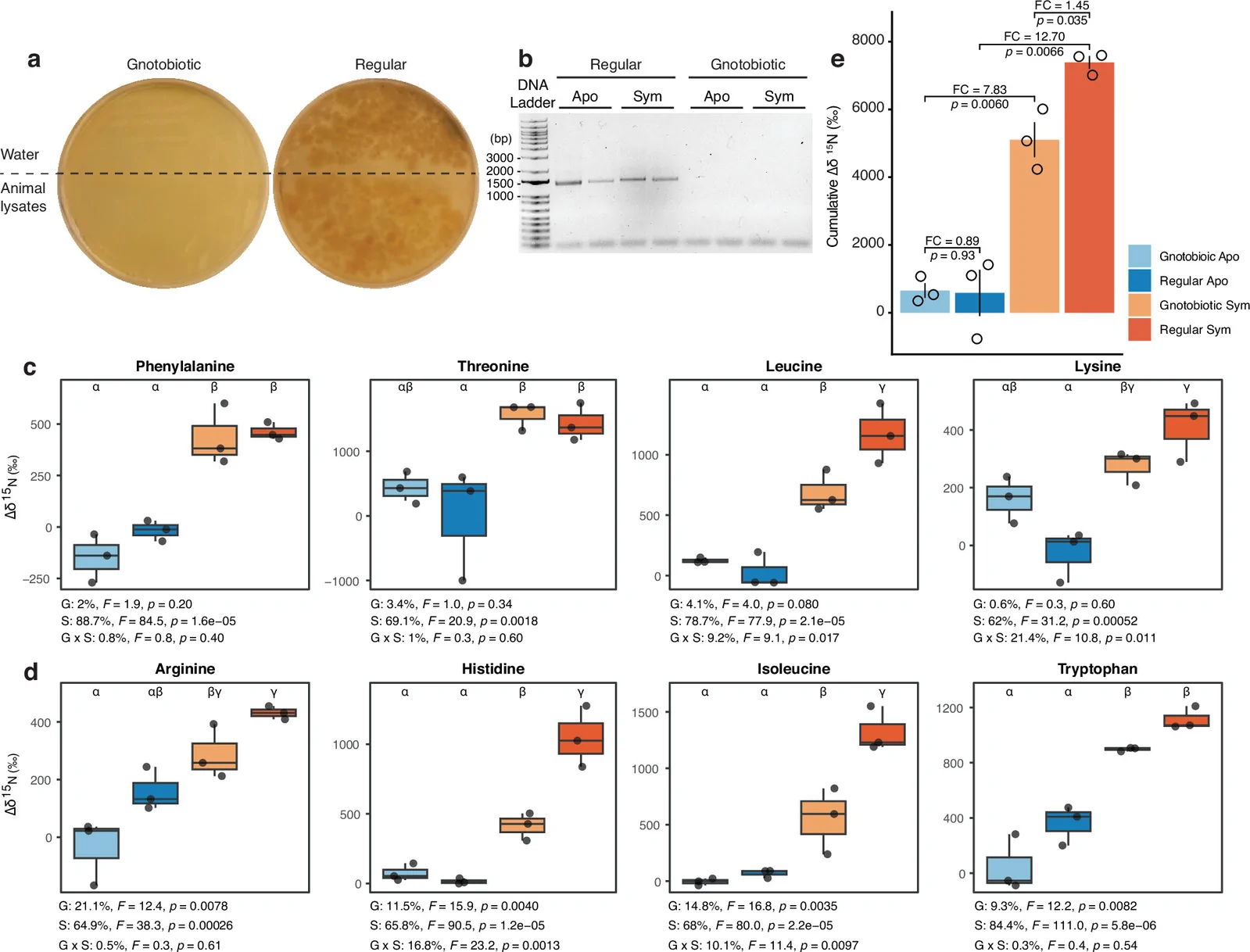
Microbial contributions to essential amino acid biosynthesis. Bacterial growth assessment via plating (**a**) and PCR validation (**b**) for gnotobiotic and regular sea anemones used in the ^15^N-nitrite supplementation experiments. In panel **b**, the leftmost lane shows the 1 kb Plus DNA Ladder (Invitrogen, catalog no. 10787018), with horizontal lines indicating the positions of the 3000, 2000, 1500, and 1000 bp markers; the 1500 bp marker band corresponds to the expected size of the 16S rRNA amplicon generated using primers 67 F/1492 R. Representative images are shown from independent experiments (*n* = 2 biological replicates per group); the full set of plating images is provided in Supplementary Fig. 4. ^15^N enrichment levels (Δδ^15^N) of eight identified essential amino acids across four experimental conditions: amino acids primarily influenced by algal symbiosis (phenylalanine, threonine, leucine, and lysine) (**c**); amino acids influenced by both bacterial and algal symbionts (arginine, histidine, isoleucine, and tryptophan) (**d**). **e** Cumulative ^15^N enrichment across all eight amino acids, highlighting synergistic effects of algal and bacterial symbionts on host nitrogen assimilation. Fold changes (FC) indicate differences in mean ^15^N enrichment between the compared groups. For panels **c** and **d**, data are presented as boxplots, with center lines representing the median, box bounds indicating the 25th and 75th percentiles (interquartile range, IQR), and whiskers extending to the minimum and maximum values within 1.5 × IQR. Individual data points represent biological replicates (*n* = 3 biological replicates per group). For panel **e**, data are presented as mean ± s.e.m. (*n* = 3 biological replicates per group). Statistical significance and variance partitioning for panels **c** and **d** were assessed using two-way analysis of variance (ANOVA), with different letters denoting significant differences between groups based on Tukey’s HSD post hoc test (*p* < 0.05). For panel **e**, pairwis**e** comparisons were performed using unpaired two-sided *t*-tests. Full statistical results are provided in Supplementary Data 12 and 13, and Supplementary Fig. 5. Source data are provided as a Source Data file.

Following verification of anemone microbial status (Fig. 4a), we identified eight essential amino acids, analyzed their ^15^N/^14^N isotopic profiles, and calculated their Δδ^15^N values (‰) relative to unlabeled controls to assess microbial contributions to essential amino acid biosynthesis. Technical-replicate injections of all samples showed strong concordance with the primary run (Pearson *r* = 0.968, Lin’s CCC = 0.964; Supplementary Fig. 5a), and independent analysis of the replicate data recapitulated the biological patterns reported below (Supplementary Fig. 5b, c), confirming that the results are robust to technical instrument variance.

These eight essential amino acids exhibited distinct patterns of ^15^N incorporation in response to the presence of bacteria and algal symbionts (Fig. 4c–e; Supplementary Data 12 and 13; Supplementary Fig. 5b, c). Phenylalanine, threonine, leucine, and lysine showed significantly increased ^15^N enrichment in symbiotic anemones, with limited additional effect from bacteria (Fig. 4c; Supplementary Fig. 5b; two-way ANOVA main effect of symbiotic state, adjusted *p* < 0.05). Notably, under aposymbiotic conditions these four amino acids exhibited Δδ^15^N values at or slightly below the unlabeled control baseline, consistent with no detectable net ^15^N incorporation above background and minor dilution by pre-existing unlabeled amino acid pools during the incubation. In contrast, arginine, histidine, isoleucine, and tryptophan showed significantly higher ^15^N incorporation in response to both bacterial and algal symbionts, and retained positive Δδ^15^N values even in aposymbiotic anemones, indicating a bacterial positive contribution to their biosynthesis that is further enhanced under symbiosis (Fig. 4d; Supplementary Fig. 5c; two-way ANOVA main effects, adjusted *p* < 0.05). Crucially, histidine, isoleucine, leucine, and lysine showed a synergistic effect, where the combined presence of bacteria and algal symbionts led to a greater increase in ^15^N enrichment than either factor alone (Fig. 4c, d; two-way ANOVA interaction effect, adjusted *p* < 0.05; Supplementary Fig. 5b, c). To assess the overall impact of symbiosis and microbial presence on host nitrogen assimilation, we calculated the cumulative ^15^N enrichment for all eight amino acids. Both gnotobiotic and regular symbiotic anemones showed significantly higher total ^15^N incorporation, 7.8- and 12.7-fold, respectively, compared to their aposymbiotic counterparts (Fig. 4e; unpaired two-sided *t*-tests, *p* < 0.05), with no difference between the two aposymbiotic groups (*p* > 0.05). Notably, regular symbiotic anemones exhibited the highest enrichment, further supporting the hypothesis that symbiosis-driven microbiome restructuring enhances host nitrogen assimilation through synergistic nitrogen transformations by microbial and algal symbionts.

## Discussion

Our findings reveal that symbiosis-driven restructuring of the microbiome at the tissue level enhances host metabolic capacity by aligning microbial function with host–symbiont nutrient exchanges. This tissue-compartmentalized microbiome adaptation underscores the holobiont as an integrated metabolic unit.

Microbial communities play essential roles in cnidarian holobiont health, metabolism, and environmental resilience^4,28^. While previous research has largely explored microbial interactions in compartments such as the surface mucus, the gastrovascular cavity, and the skeleton, how symbiosis influences the functional roles and spatial distribution of bacteria within host tissues has remained unclear^29,30^. Here, we resolve this gap by showing that host tissues harbor distinct microbial communities whose spatial reorganization in response to symbiosis functionally contributes to holobiont nitrogen assimilation and essential amino acid synthesis.

By integrating tissue-specific metatranscriptomics with stable isotope labeling and high-resolution metabolomics, our findings reveal spatial microbial organization at the tissue level^29,31,32^, showing differential distribution and function of the bacterial community across gasterodermis and epidermis in response to symbiosis.

Metatranscriptomic analyses revealed that symbiosis-driven restructuring not only affects taxonomic composition, but also functional shifts in gene expression, particularly in carbon, nitrogen, amino acid, and vitamin metabolism — key processes that are tightly interconnected within holobiont nutrient cycling. Given that nitrogen is typically a limiting nutrient in oligotrophic coral reef ecosystems, efficient nitrogen acquisition and assimilation are critical for sustaining holobiont metabolism. However, nitrate and nitrite are inaccessible to the cnidarian host, and their assimilation via microbial partners is energetically costly as the processes rely on substantial reducing power and carbon sources^33^, making them highly dependent on energy resources available in symbiotic tissues. Interestingly, only nitrite reductase (NiR) genes, specifically nasB and nasD, which function in assimilatory pathways, were upregulated, whereas nitrate reductases were not detected. This pattern may reflect oxygen-sensitive regulatory mechanisms known to repress the expression of nitrate reductases under oxygen-rich conditions during the day^34^, when nitrite assimilation by NiR becomes more favorable due to photosynthetic carbohydrate availability. The upregulation of nitrite reductase in the symbiotic gastrodermis further indicates that microbial nitrogen assimilation is fueled by the increased availability of photosynthetically derived carbon^35,36^. This pattern complements the host’s spatial metabolic responses and aligns with the symbiosis-induced upregulation of gastrodermal glucose and ammonium transporters that we previously characterized^3^. This direct metabolic link between bacterial nitrogen processing and host-algal interactions challenges the traditional host-algae-centric view of nutrient exchange, instead emphasizing the holobiont as a metabolically coordinated unit^37,38^.

Our isotope tracing experiment provides further direct evidence that symbiosis restructures the microbiome in a way that allows it to contribute to holobiont amino acid biosynthesis. Microbial assimilation of nitrite resulted in significant incorporation of ^15^N from into essential amino acids, depending on bacterial and symbiotic states. Strikingly, certain amino acids (e.g., histidine, isoleucine, leucine, lysine) showed a synergistic response, where combined microbial and algal symbiont presence resulted in significantly higher ^15^N enrichment than either alone. This suggests that microbial nitrogen processing actively supplements host metabolism^39–42^, potentially buffering against nutrient fluctuations and reinforcing holobiont metabolic flexibility^28^.

Unlike denitrification, which has been widely studied in coral holobionts, nitrite reduction provides a direct mechanism for transforming abundant nitrogen species not directly accessible to the host into more bioavailable forms. This microbial-mediated nitrogen remodeling enhances holobiont nitrogen availability and retention, influencing host-algal interactions and potentially regulating symbiont density by modulating nitrogen availability^43–46^. Specifically, microbes may function as metabolic gatekeepers, regulating the flux of inorganic nitrogen sources—accessible to algal symbionts but beyond host biochemical capabilities—thereby contributing to symbiotic nutrient balance and homeostasis.

Our findings suggest that microbial shifts in response to symbioses are not incidental but a core feature of holobiont organization, reflecting an adaptive optimization of nutrient cycling that promotes functional synergies. The presence of taxa such as *Bacillus subtilis* and *Klebsiella oxytoca*, known for their probiotic functions and stress resilience in other host systems, suggests that symbiotic microbiomes may be selectively enriched for traits that help buffer the holobiont against environmental perturbations. These microbes have been implicated in nitrogen metabolism, vitamin synthesis, niche occupation, and microbiome modulation^24–27^, and are even actively explored in coral probiotic formulations to mitigate bleaching-related stresses^47–49^. By integrating microbial nitrogen transformations into host metabolism and converting nitrogen forms inaccessible to the host into essential amino acids, the microbiome restructuring actively contributes to the holobiont metabolic flexibility and resilience. This aligns with emerging concepts of microbiome plasticity in cnidarians, where microbial community shifts support nutrient homeostasis, symbiotic regulations, and potentially improved resilience to stressors such as thermal fluctuations, nutrient enrichment, or bleaching^7,32,50^. Conversely, disruptions in nutrient exchange and microbial nitrogen cycling during heat stress or bleaching may compromise this metabolic synergy, contributing to holobiont destabilization and dysbiosis.

While this study provides high-resolution mechanistic insights into the functional restructuring of the cnidarian microbiome, we acknowledge certain experimental limitations. First, although our gnotobiotic anemones were rigorously validated using both culture-dependent and molecular (16S rRNA PCR) approaches, the inherent challenges of detecting extremely low-abundance or unculturable taxa mean that the presence of a trace residual microbiome cannot be entirely ruled out. Additionally, due to the severely depleted microbial biomass in these treatments, we did not perform metatranscriptomics on the gnotobiotic samples. While our ^15^N stable isotope tracking effectively decoupled host and microbial metabolic contributions, future studies incorporating RNA-seq on gnotobiotic holobionts would be invaluable for further isolating host-specific transcriptomic responses. Second, our active microbiome profiling is primarily presented at higher taxonomic ranks. Fine-scale taxonomic assignments in marine samples is method-sensitive and constrained by current database coverage. However, the functional shifts we report, particularly in nitrogen and carbon metabolism, are consistent across the taxonomic profiling approaches we used and are biologically informative. Finally, while Aiptasia serves as a tractable model for isolating the fundamental mechanisms of cnidarian-dinoflagellate symbiosis, its lack of a calcified skeleton presents a notable functional divergence from reef-building corals. The calcium carbonate skeleton of scleractinian corals introduces additional anatomical and physical niches, such as endolithic microenvironments, and imposes unique energetic demands.

Although demonstrated here in a cnidarian model, our findings suggest a broader biological principle: host-associated microbiomes may adaptively restructure within tissue compartments to complement host metabolism in nutrient-limited environments. Such compartmentalized microbial function could represent a widespread strategy in animal host–microbe symbioses, where microbial metabolic plasticity enhances holobiont performance and resilience during environmental fluctuations.

To fully resolve how these synergistic metabolic networks operate within the structural complexity of true reefs, future studies should apply multi-omics and isotopic approaches to diverse, calcifying coral species. Specifically, pairing our amino-acid-level ^15^N tracing with simultaneous ^13^C measurements of the same amino acids would directly resolve whether the carbon backbones of microbially supported essential amino acids originate from algal photosynthates or other carbon pools, providing a more complete picture of the metabolic interdependencies described here. Furthermore, it will be critical to examine how microbial nitrogen cycling responds to environmental stressors such as ocean warming and eutrophication, and whether microbial metabolic plasticity contributes to long-term holobiont stability. Integrating these highly resolved spatial and functional microbiome dynamics into broader holobiont models will ultimately refine our understanding of how microbial interactions shape coral reef function and resilience in a changing ocean.

## Methods

### Animals and maintenance

All experiments in this study were conducted using the sea anemone Aiptasia (sensu *Aiptasia pallida*, *Exaiptasia pallida*, or *Exaiptasia diaphana*) clonal strain CC7^51^. Anemones were maintained in autoclaved, filtered seawater from the Red Sea (hereafter referred to as “seawater”).

To generate anemones free of algal endosymbionts (aposymbiotic), individuals underwent a cold shock treatment at 4 °C for 4 h, followed by ~30 d of exposure to 50 μM Diuron (Sigma-Aldrich) with daily water changes^46^. The absence of algal symbionts was confirmed by fluorescence microscopy. To establish the symbiotic line used in this study, a batch of these aposymbiotic anemones was subsequently inoculated with *Breviolum minutum* strain SSB01^52^. These newly inoculated anemones were communally cultured in polycarbonate tanks for ~8 weeks to ensure the establishment of a stable symbiont population. Symbiosis stability was assessed via the development of uniform brown host tissue coloration, indicative of high symbiont densities, as repopulation typically reaches stable densities within 4 weeks. Both the symbiotic and the parallel aposymbiotic anemones used in the subsequent experiments were sourced from these established, long-term laboratory stocks.

Both symbiotic and aposymbiotic anemones were maintained under a 12 h:12 h light:dark cycle with ~40 μmol photons m^–2^s^–1^ of photosynthetically active radiation. Anemones were fed with freshly hatched brine shrimp (*Artemia salina*) approximately three times per week, with seawater changes performed the day after feeding.

For tissue-specific RNA-seq experiments, eight 500-ml tanks were set up, with four tanks for symbiotic (Sym) and four for aposymbiotic (Apo) anemones. Each tank contained anemones of similar size (pedal disc ~0.5 cm in diameter) and was maintained in the same incubator throughout the study. To minimize potential contamination from food sources, anemones were not fed for three days prior to sampling. Apo anemones were confirmed to be free of algae by fluorescence microscopy immediately before sampling.

### Laser microdissection

A total of eight anemones (one from each of the eight RNA-seq experimental tanks: *n* = 4 Sym, *n* = 4 Apo) were collected at 11:00 AM (6 h into the light phase), snap-frozen in liquid nitrogen, embedded in Tissue Freezing Medium (Electron Microscopy Sciences), and stored at –80 °C until cryosectioning. Sample blocks were equilibrated for 20 min in a pre-chilled cryostat (CM3050 S, Leica) set to –23 °C, and sectioned at a thickness of 8 μm. Sections were transferred onto pre-cooled RNase-free polyester membrane metal frame slides (Leica) and air-dried for 5 min in an RNase-free fume hood.

Gastrodermal (Gas) and epidermal (Epi) tissue layers were identified in sections, primarily from tentacles, at both 10× and 20× magnifications using a Leica LMD 6000 microscope with Leica filter cubes B/G/R and A (Supplementary Fig. 1). Regions of interest (ROIs) were traced using the laser-microdissection (LMD) software and dissected with an ultraviolet laser beam. To collect a total tissue area of approximately 2–3 mm^2^ per tissue type from each animal, ~30–50 ROIs were dissected for gastrodermis, which typically presented as larger, continuous areas, and ~40–80 ROIs for the thinner epidermis. Dissected tissue fragments were collected into CapSure Macro LCM Caps (Thermo Fisher) containing 40 μl RNA-extraction buffer from the Arcturus PicoPure RNA Isolation Kit (Thermo Fisher). The harvested samples were lysed at 42 °C for 30 min, vortexed briefly, and stored at –80 °C until RNA extraction.

### RNA extraction and sequencing

Total RNA was extracted from LMD-isolated samples using the Arcturus PicoPure RNA Isolation Kit, following the protocol for use with CapSure Macro LCM Caps. RNA quality was assessed using an Agilent 2100 Bioanalyzer with the Agilent RNA 6000 Pico Kit. cDNA was synthesized using the Ovation RNA-Seq System V2 kit (NuGen) with a combination of random hexamers and a poly-T chimeric primer. Amplified cDNA was sheared to ~200 bp using a Covaris ultrasonicator, following the manufacturer’s recommended protocol. Sequencing libraries, with a mean insert size of 200–250 bp, were prepared using the NEBNext Ultra II DNA Library Prep Kit for Illumina sequencing. Libraries were pooled and sequenced on four lanes of an Illumina HiSeq 2000 to generate paired-end reads. Per-sample sequencing quality metrics, including raw read counts, post-filtering depth, and rRNA/mRNA partitioning, are provided in Supplementary Data 1.

### rRNA analysis

RNA-seq reads were processed using Trimmomatic v0.32 to remove adapter sequences and low-quality bases^53^. Specifically, adapters were removed using the ILLUMINACLIP parameter. Read ends were trimmed if quality scores fell below 3 (LEADING:3, TRAILING:3), and a sliding window approach was applied to truncate reads when the average quality within a 4-base window dropped below a Phred score of 20 (SLIDINGWINDOW:4:20). Finally, any reads that fell below a minimum length of 36 base pairs after trimming were discarded (MINLEN:36). The resulting pair-end reads were error-corrected with SPAdes v3.9.0^54,55^. rRNA sequences were identified and extracted from the error-corrected reads using SortMeRNA v2.1b^56^.

For bacterial community analyses, 16S rRNA transcripts were assembled using Pandaseq v2.11 with parameters -t 0.32 -A pear -B^57^. Assembled transcripts were filtered to retain high-quality sequences with a minimum length of 100 bp, a maximum homopolymer of 6, and no ambiguous bases. Taxonomic classification of the processed 16S rRNA gene sequences was carried out using the Wang naïve Bayesian classifier method (80% bootstrap confidence threshold) in MOTHUR v39.5, with the GreenGenes database v13_8 as a reference^58–60^.

### Metatranscriptome assembly and classification

To assess the functional potential of bacterial communities, protein-coding transcripts were assembled from non-rRNA reads retained by SortMeRNA using Trinity v2.2.0 with the parameter --min_contig_length 300^61^. Coding regions within the assembled transcripts were identified using Transdecoder v2.0.1 (https://github.com/TransDecoder/). Transcripts encoding proteins longer than 100 amino acids and containing conserved domains from the Pfam database were retained for downstream analyses.

Protein-coding genes from all samples were combined into a comprehensive dataset and clustered into a non-redundant gene catalog using cd-hit v4.8.1^62,63^. Clustering was performed with a global sequence identity threshold of 95% and an alignment coverage of at least 80% over the shorter gene sequence. The resulting non-redundant gene catalog was annotated using KMAP^64^, which integrates functional prediction from multiple databases^65^, including the KEGG Orthology database^66^. Annotation criteria included a BLAST score cutoff of 70 for functional assignment and taxonomic classification using the lowest common ancestor approach based on the NCBI nomenclature. The relative abundance of bacterial, archaeal, and viral transcripts across samples and the phylum-level bacterial mRNA composition were computed from this annotated non-redundant gene catalog.

### Complementary taxonomic profiling

To extend taxonomic resolution beyond the phylum and order levels captured by the 16S rRNA analysis and the KMAP-based metatranscriptome classification, we performed two additional classification analyses targeting complementary fractions of the sequencing data. To profile the transcriptionally active (mRNA-based) community, total sequenced reads were processed through MetaPro v3.0.1^67^, a dedicated metatranscriptomics pipeline that performs integrated read quality filtering, host removal, rRNA depletion, and taxonomic and functional annotation of the resulting mRNA fraction. To profile the ribosomal (rRNA-based) community, the full rRNA pool extracted by SortMeRNA was independently classified using Kraken2 v2.1.3 against the PlusPF pre-built database (build 2024-09-04) with default parameters^68^. Together, these analyses provided complementary higher-resolution views of the same samples: the mRNA community via MetaPro and the rRNA community via Kraken2 (Supplementary Data 2).

Alpha diversity metrics (observed genera, Shannon, Simpson) were computed per sample and tested for main effects of tissue layer, symbiotic state, and their interaction using two-way ANOVA followed by Tukey’s honest significant difference (HSD) post hoc tests for pairwise group comparisons. Beta diversity was assessed with Bray–Curtis dissimilarities on family- and genus-level abundance tables, and differences between groups were evaluated using two-way PERMANOVA (vegan::adonis2; ~ Symbiosis × Tissue; 9,999 permutations), with PERMDISP (vegan::betadisper) used to confirm homogeneity of within-group dispersion (Supplementary Fig. 2; Supplementary Data 3).

### Differential expression and functional analysis

To further characterize how symbiosis and tissue compartmentalization reshape the functional activity of the microbiome, we performed differential expression and pathway-level analyses on the annotated metatranscriptome. For these analyses, only transcripts classified as microbial, specifically bacteria and archaea, were considered. Transcript abundances were quantified using kallisto v0.43.0^69^ and processed with sleuth v0.27.0^70^. Filtered, library-size-normalized TPM values were extracted via sleuth and used as input for both differential expression and ordination analyses (Supplementary Data 4).

To first assess global variation in microbial gene expression across all 16 samples simultaneously, we performed a principal coordinates analysis (PCoA) based on sample-to-sample distances computed from the filtered, normalized expression matrix (Supplementary Fig. 3). Distances were calculated using both Bray–Curtis dissimilarity on normalized TPM values and Euclidean distance on log_2_(TPM + 1)-transformed TPM values. The contributions of symbiotic state, tissue layer, and their interaction to overall expression variance were quantified by two-way PERMANOVA (vegan::adonis2; ~ Symbiosis × Tissue; 9999 permutations), with within-group dispersion homogeneity confirmed using PERMDISP (vegan::betadisper).

Building on this global view, differential gene expression was assessed for four pairwise comparisons: ApoGas vs. ApoEpi and SymGas vs. SymEpi to resolve tissue effects within each symbiotic state, and SymEpi vs. ApoEpi and SymGas vs. ApoGas to resolve symbiosis effects within each tissue layer (Supplementary Data 5). For each comparison, sleuth was prepared on the relevant subset of samples with a single-factor design ( ~ condition), fitted, and tested using a Wald test on the corresponding coefficient. Genes with a Benjamini–Hochberg-corrected *p*-value (*q*-value) <0.05 were considered significantly differentially expressed. To visualize variation within each of these four contrasts, principal component analysis (PCA) of microbial gene expression was performed using the *prcomp* function (R *stats* package), with genes mean-centered and scaled to unit variance, separately for each of the four pairwise sample subsets corresponding to the differential expression contrasts.

To infer the functional roles of differentially expressed transcripts, gene ontology (GO) term enrichment analyses were performed using topGO v2.52.0 on the biological process ontology, using the weight01 algorithm with Fisher’s exact test as the underlying statistic^71^. For each pairwise comparison, significantly differentially expressed microbial transcripts were tested as the gene set of interest against a background of all annotated expressed microbial transcripts in the metatranscriptome. The weight01 algorithm accounts for the hierarchical structure of the GO graph by down-weighting parent terms whose enrichment is driven by more specific child terms. Consistent with the recommendation of the developers, no further multiple-testing correction was applied, as the resulting p-values are not independent classical Fisher *p* values. GO terms with *p* < 0.05 were considered significantly enriched (Supplementary Data 6).

Microbial activity analyses were conducted on KEGG pathways annotated based on the metatranscriptome, using gene set variation analysis (GSVA; Supplementary Data 7), as previously described^44,72^. Pathway activity, represented by GSVA scores, was clustered and visualized using ComplexHeatmap v2.21.2^73^. The effects of tissue layers and symbiotic states on pathway activities were tested using a generalized linear model implemented in limma v3.60.3 (Supplementary Data 8)^74^.

### Gnotobiotic Aiptasia generation

Bacteria-depleted anemones were generated following an established protocol^75^. To generate source populations for the downstream isotope labeling experiments, four glass beakers were set up, each containing 30 Aiptasia polyps with similar size (pedal disc diameter ~0.5 cm): two beakers for symbiotic and two for aposymbiotic anemones. One beaker of each symbiotic state was subjected to the gnotobiotic treatment, while the other served as the regular control. Briefly, anemones designated for the gnotobiotic treatment were washed repeatedly with seawater and then incubated for 15 min in an antibiotic solution consisting of 50 μg/ml each of Carbenicillin, Chloramphenicol, Rifampicin, and Nalidixic acid in seawater. Following incubation, anemones were transferred into sterilized glass beakers containing fresh antibiotic solution, where they were maintained without feeding for 7 days, with daily media changes. After the 7-day treatment, anemones were transferred to autoclaved 0.22-μm-filtered seawater and allowed to recover for 24 h.

To verify bacterial depletion at the end of the treatment, four anemones were randomly sampled from each of the four beakers. Two anemones per beaker were used for culture-based validation by plating tissue homogenates and corresponding beaker seawater samples (20 μl) on marine agar plates. The absence of bacterial growth after a 3-d incubation at 25 °C confirmed the depletion of culturable bacteria (Supplementary Fig. 4). To account for unculturable bacteria, the remaining two anemones per beaker were homogenized and subjected to total DNA extraction using the AllPrep PowerViral DNA/RNA Kit. PCR amplification targeting the bacterial 16S rRNA gene was performed using universal primers 67 F (5’-CAGGCCTAACACATGCAAGTC-3’) and 1492 R (5’-GGTTACCTTGTTACGACTT-3’), synthesized by Integrated DNA Technologies (IDT). The absence of detectable PCR products on an agarose gel was consistent with effective bacterial depletion to below detection limits in the antibiotic-treated anemones.

### Isotope labeling and metabolite extraction

Following verification of the gnotobiotic state, the isotope labeling experiment was set up with eight different conditions: gnotobiotic symbiotic, gnotobiotic aposymbiotic, regular symbiotic, and regular aposymbiotic anemones incubated in either ambient seawater (control) or seawater amended with 30 μM ^15^N-nitrite. This concentration is consistent with established cnidarian stable isotope tracing protocols^76–78^, providing a robust signal for detecting downstream metabolite synthesis without inducing toxicity. Three individual anemones (*n* = 3 biological replicates) from the appropriate source beakers were assigned to each of the eight conditions, yielding a total of 24 anemones. The anemones were incubated for the -2-week to allow sufficient time for the de novo synthesis and cross-compartmental translocation of amino acids. During this period, anemones were maintained under a 12 h:12 h light:dark cycle ( ~ 40 μmol photons m^–2^s^–1^) and were starved to prevent isotopic dilution of the ^15^N signal by exogenous food sources. Tracer concentration were maintained via complete water and treatment changes every 48 h.

After the incubation, amino acids were extracted from the animal tissue as previously described^79^. Briefly, animal tissues were homogenized using micro-pestles (Thermo Fisher) in a lysis buffer consisting of 0.2 M Tris-HCl (pH 7.5), 0.5% Triton-X, and 2 M NaCl. The homogenates were sheared repeatedly through a 25 G needle to release algal symbionts, then centrifuged at 10,000 × *g* for 5 min at 4 °C. Pellets containing symbionts were discarded, and supernatants containing animal tissue lysates were transferred to fresh tubes on ice. The same centrifugation process was repeated three times to ensure complete removal of symbionts. Representative supernatants from the final centrifugation were examined by fluorescence microscopy to confirm the absence of algal chlorophyll.

Symbiont-free animal tissue lysates were snap-frozen in liquid nitrogen, and metabolites were extracted using a freeze-drying method. Briefly, lysates were further diluted in 5 mL milli-Q water and lyophilized using a freeze dryer (Labconco). Lyophilized samples were resuspended in 1 mL of pre-chilled ( − 20 °C) 100% methanol, sonicated for 30 min at 4 °C in an ultrasonication bath (Branson), and centrifuged at 3000 × *g* for 30 min at 4 °C. The resulting supernatants were collected, while pellets were resuspended in 1 mL of pre-chilled 50% methanol and centrifuged at 3000 × *g* for 30 min at 4 °C. Supernatants from both steps were combined, centrifuged at 16,000 × *g* for 15 min at 4 °C to remove particulates, and dried using a speed vacuum concentrator (Labconco). The final extracts were stored at −80 °C until further analysis.

### Ultra-high-performance liquid chromatography high-resolution mass spectrometry (UHPLC-HR-MS)

Amino acid standards were prepared by diluting Amino Acid Standard H (Thermo Fisher) to final concentrations of 25 μM for all amino acids, except for L-cystine (12.5 μM), followed by a 10-fold dilution in 25% aqueous methanol. Metabolite extracts from animal tissues were resuspended in 200 μL of 25% aqueous methanol, filtered through 0.2 μm filters, and subjected to UHPLC-HR-MS analysis.

Amino acid separation and detection were performed using a Dionex Ultimate 3000 UHPLC system coupled to a Q Exactive Plus mass spectrometer (Thermo Fisher) equipped with a heated electrospray ionization source. Chromatographic separation was conducted on an ACQUITY UPLC® BEH Amide column (130 Å, 1.7 μm, 2.1 mm × 100 mm, Waters) maintained at 35 °C. The mobile phases A (water/formic acid, 100/0.1, v/v) and B (acetonitrile/formic acid, 100/0.1, v/v) were employed for eluting amino acids at a flow rate of 0.25 mL/min and with the following gradient program: 0–8 min, 95% B to 25% B; 8–11 min, 25% B; 11–12 min, 25% B to 95% B; 12–15 min, 95% B. Injection volumes were set at 2 μL. Mass spectrometric detection was conducted in positive mode with a spray voltage of 3.0 kV, a sheath gas flow rate of 35 arbitrary units, an auxiliary gas flow rate of 10 arbitrary units, a spray capillary temperature of 300 °C, an auxiliary gas heater temperature of 325 °C, an automatic gain control (AGC) target of 3 × 10^6 ^ions, and a resolution of 280,000. Data acquisition, peak identification, signal extraction, and quantification were performed using Xcalibur v1.6 (Thermo Fisher).

All sample extracts were analyzed in duplicate using two consecutive injections on the same analytical day, providing a primary run and a paired technical replicate to assess instrument variability. The primary run was used for the differential ¹⁵N enrichment analyses presented in the main figures. The technical replicate was independently processed and quantified using the same Xcalibur workflow, and the agreement between the two injections was assessed across all 192 paired measurements (4 experimental conditions × 8 essential amino acids × 2 treatments × 3 biological replicates) using Pearson correlation and Lin’s concordance correlation coefficient (CCC). The results of this technical replicate validation are provided as Supplementary Fig. 5.

### Metabolite identification and quantification

Amino acids were identified and assigned based on their accurate mass and retention time, which were verified using appropriate standards. Isotopologues of each amino acid were extracted and quantified based on their peak areas. Abundances of isotopologues from control samples incubated with ambient seawater were used as baseline values. Ratios of ^15^N-containing isotopologues (e.g., ^12^C^15^N) relative to their natural, unlabeled forms (^12^C^14^N) were calculated and normalized prior to the following statistical evaluations.

### Statistical analysis

All statistical analyses and data visualizations were conducted in R, with the complete analytical pipeline and source code available at GitHub: https://github.com/1cuigx/tissue-specific-microbiome. Assumptions of normality and homogeneity of variance were assessed using Shapiro–Wilk and Levene’s tests, respectively (Supplementary Data 9–13). To meet these criteria, relative abundance percentages were arcsine square root transformed prior to analysis. For variables that deviated from normality, non-parametric Kruskal–Wallis tests with Dunn’s post hoc comparisons were performed; these yielded results concordant with parametric models, confirming the robustness of the findings (Supplementary Data 9–13).

Differences in relative microbial abundances and individual ^15^N enrichment levels were evaluated via two-way ANOVA, with variance partitioning derived from the models’ sum of squares. Cumulative ^15^N enrichment was compared using unpaired two-sided *t*-tests. Significant ANOVA effects were further examined using Tukey’s HSD post hoc tests. To account for multiple comparisons across taxa and metabolites, all *p*-values were adjusted using the Benjamini-Hochberg false discovery rate (FDR) procedure. Detailed statistical summaries are available in Supplementary Data 9–13.

### Reporting summary

Further information on research design is available in the Nature Portfolio Reporting Summary linked to this article.

## Supplementary information

Supplementary Information

Description of Additional Supplementary Files

Supplementary Datasets 1–13

Reporting Summary

Transparent Peer Review file

## Source data

Source Data

## Data Availability

Raw data generated from UHPLC-HR-MS analysis have been deposited in the NIH Common Fund’s National Metabolomics Data Repository under Project ID PR002710 [https://doi.org/10.21228/M8C25W]. RNA-seq data generated in this study have been deposited in the NCBI Sequence Read Archive under BioProject accession code PRJNA631577. The analysis code and input data required to reproduce the main and supplementary display items are provided in the associated GitHub repository [https://github.com/1cuigx/tissue-specific-microbiome]. All other relevant data are provided in the Supplementary Data. Source data are provided with this paper.

## Code availability

All statistical analyses and data visualizations were conducted in R, with the complete analytical pipeline and source code available at GitHub: https://github.com/1cuigx/tissue-specific-microbiome. The repository has been archived in Zenodo and is available at https://doi.org/10.5281/zenodo.22806098^80^.
