# Supplementary material for: Algal symbiosis adaptively restructures the cnidarian microbiome to enhance holobiont metabolism
Source: Nat Commun. 2026 Nov 2;17:11128. doi: 10.1038/s41467-026-79238-6 (PMC13728110; doi:10.1038/s41467-026-79238-6)
Supplement: Supplementary file 1 [file 41467_2026_79238_MOESM1_ESM.pdf]

Supplementary Information for

Algal symbiosis adaptively restructures the cnidarian microbiome to enhance holobiont metabolism

Guoxin Cui[1,2]†*, David K. Ngugi[3]†, Migle Konciute[2], Si Cheng[4], Kit Xi Liew[5], Jianing Mi[4], Salim Al-Babili[5,6], Manuel Aranda[2]*

*Corresponding author. Guoxin Cui (guoxincui@cuhk.edu.hk) and Manuel Aranda (manuel.aranda@kaust.edu.sa)

This PDF file includes:

Supplementary Figures 1 to 5

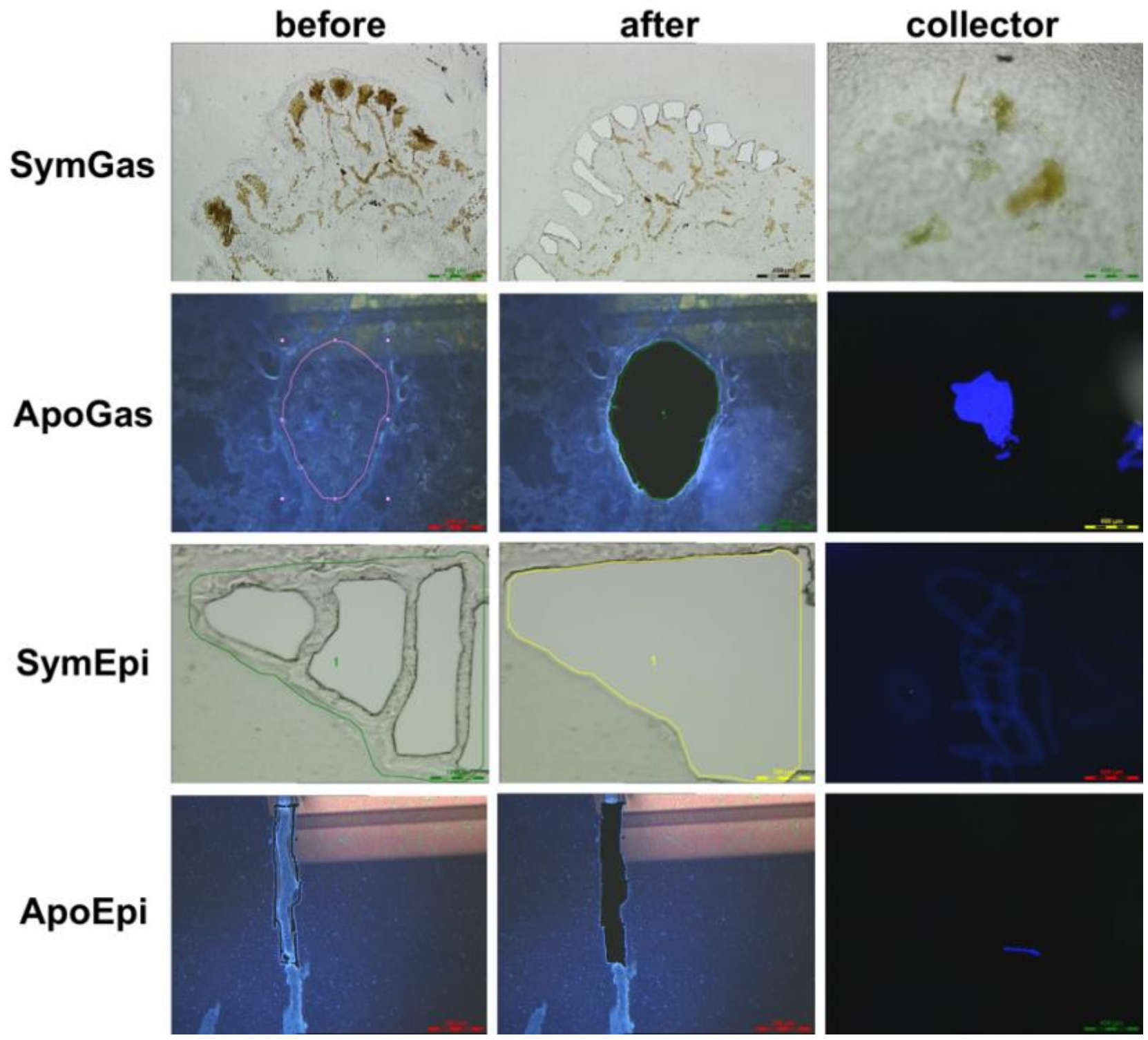

Supplementary Fig. 1: Representative images of the laser microdissection process demonstrating the isolation of gastrodermal and epidermal tissues.

Symbiotic samples were imaged under white light, whereas aposymbiotic samples were visualized using a Leica filter cube B/G/R or A, which helped in identifying their epidermal cell layer. Sym, symbiotic; Apo, aposymbiotic; Gas, gastrodermis; Epi, epidermis. Reproduced from (Cui et al., 2023) [1].

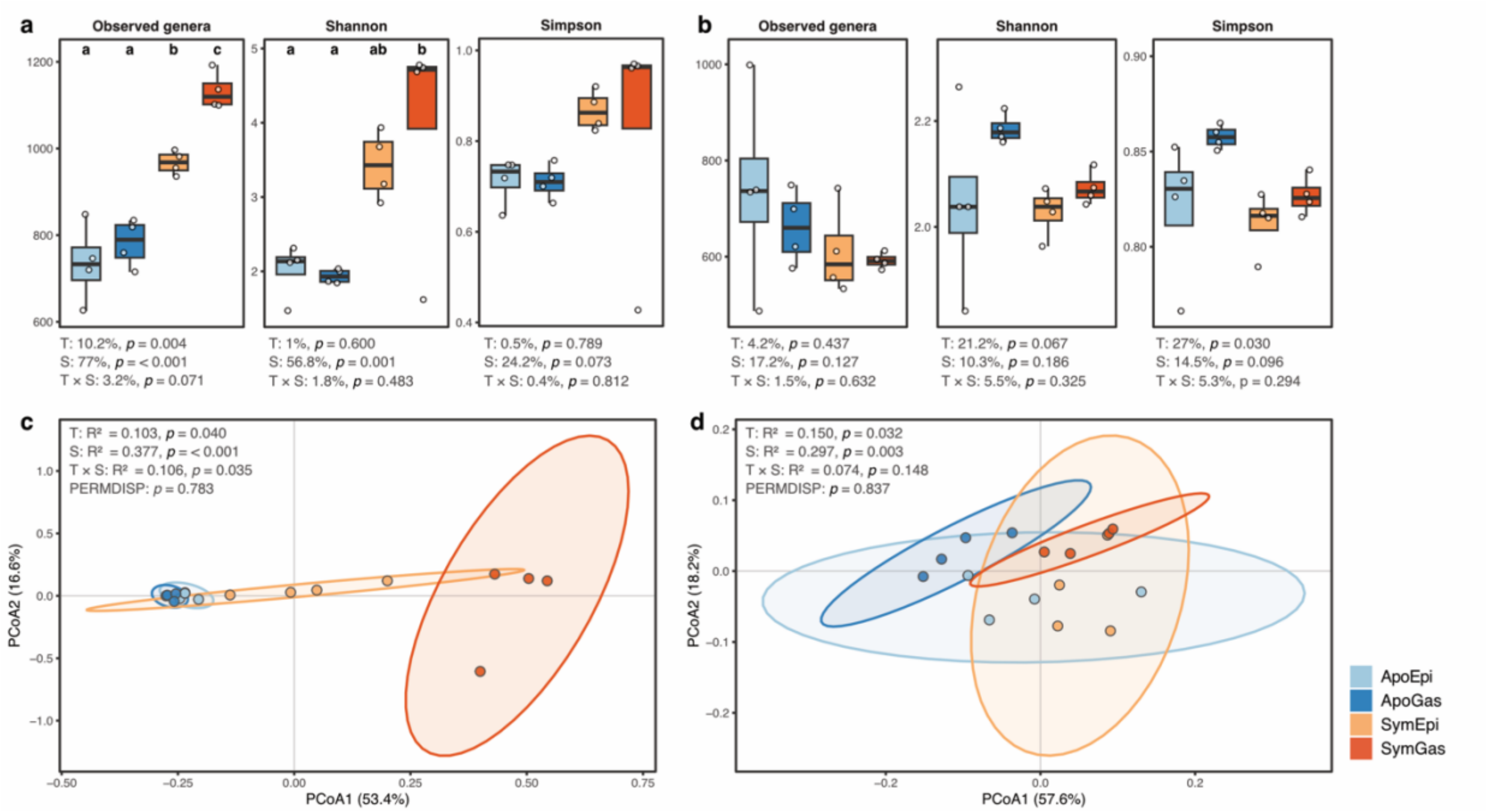

Supplementary Fig. 2: Genus-level alpha and beta diversity of mRNA- and rRNA-based bacterial communities

a,b, Alpha diversity of the mRNA-based community profiled by MetaPro (**a**) and the rRNA-based community profiled by Kraken2 (**b**), shown as observed genera, Shannon, and Simpson indices across the four groups. Boxplots show the median (center line), interquartile range (box bounds), and individual biological replicates (n = 4 biologically independent samples per group). Two-way ANOVA results for tissue layer (T), symbiotic state (S), and their interaction (T × S) are indicated in each facet; different letters denote significant differences among groups based on Tukey's HSD post hoc test ($p < 0.05$). **c,d,** Principal coordinates analysis (PCoA) of Bray–Curtis dissimilarities based on genus-level MetaPro (**c**) and Kraken2 (**d**) profiles, with 95% confidence ellipses for each group. Two-way PERMANOVA statistics (vegan::adonis2; ~Symbiosis × Tissue; 9,999 permutations) for tissue layer, symbiotic state, and their interaction, together with PERMDISP p-values (vegan::betadisper), are shown in the plots. Full statistical results are provided in **Supplementary Data 3**.

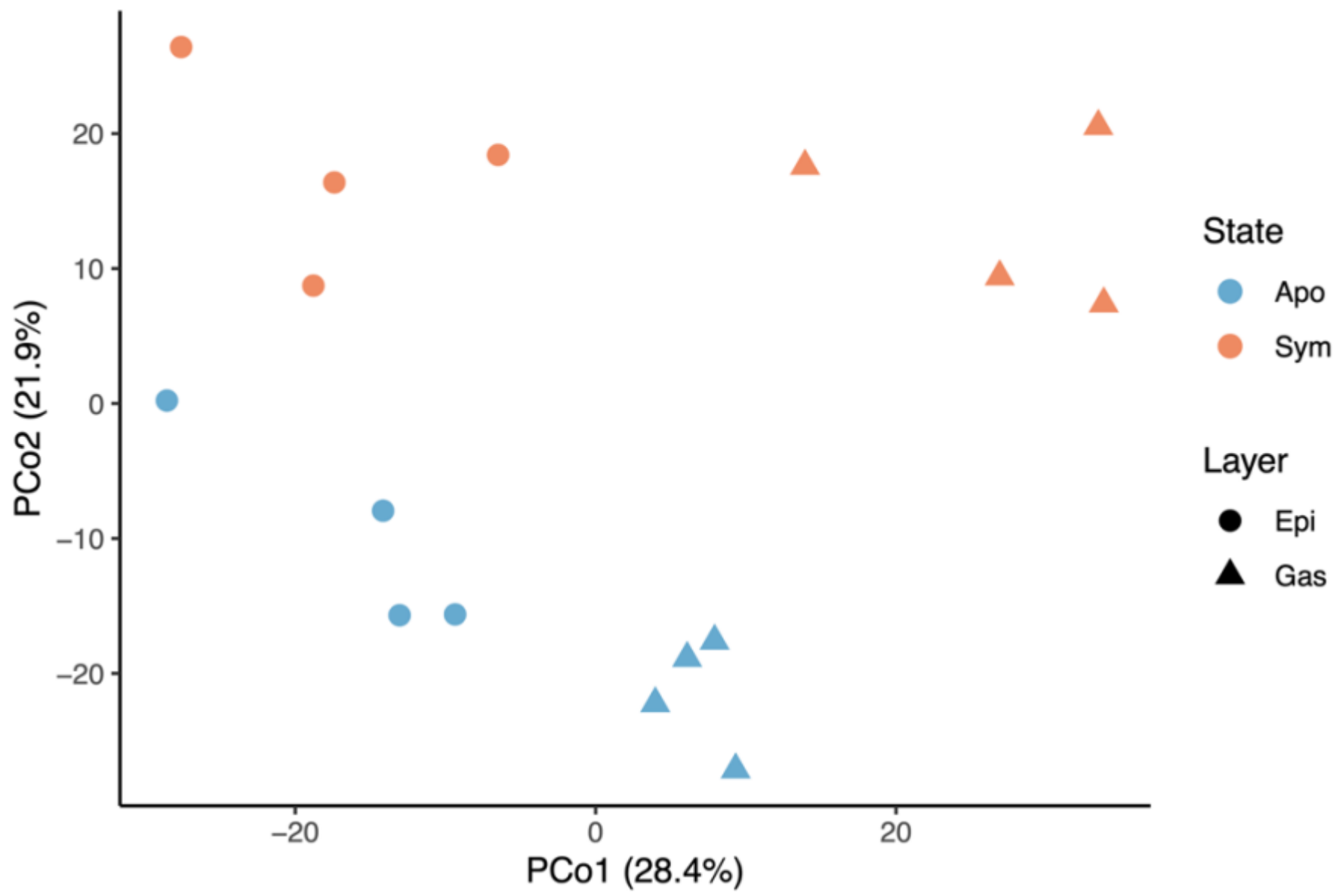

Supplementary Fig. 3: Global ordination of microbial gene expression across all 16 samples.

Principal coordinates analysis (PCoA) based on log-Euclidean distances of $\log_2$(TPM + 1)-transformed transcript abundances. Each point represents one biological replicate (n = 4 biologically independent samples per group), colored by symbiotic state (Apo, aposymbiotic; Sym, symbiotic) and shaped by tissue layer (Epi, epidermis; Gas, gastrodermis). Two-way PERMANOVA (~ State × Layer; vegan::adonis2; 9,999 permutations) confirmed significant main effects of both symbiotic state ($R^2 = 0.20$, $p = 0.0001$) and tissue layer ($R^2 = 0.23$, $p = 0.0001$), with a marginal interaction term ($R^2 = 0.08$, $p = 0.051$). PERMDISP confirmed homogeneous within-group dispersions for both factors (State $p = 0.56$; Layer $p = 0.75$).

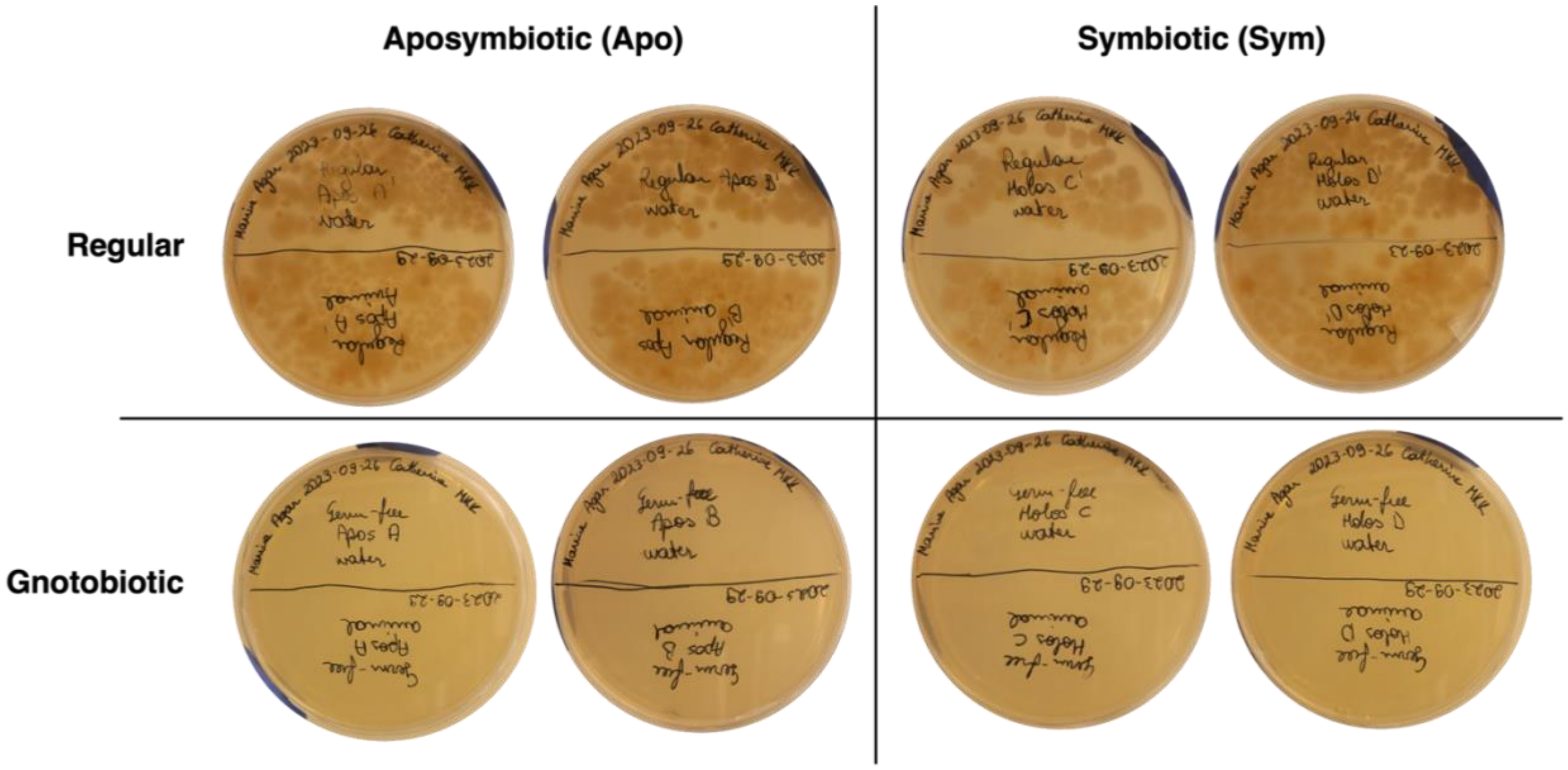

Supplementary Fig. 4: Culture-based verification of bacterial depletion in gnotobiotic anemones. Marine agar plates inoculated with tissue homogenates (bottom half of each plate) and corresponding beaker seawater samples (top half of each plate; 20 µL each) collected at the end of the antibiotic treatment, photographed after 3 days of incubation at 25 °C. Top row: plates from regular (non-treated) anemones, showing extensive bacterial growth from both tissue and seawater samples. Bottom row: plates from gnotobiotic (antibiotic-treated) anemones, showing no detectable bacterial colonies from either tissue or seawater samples. Representative images from these plating experiments are shown in **Fig. 4a**. Complementary molecular validation of bacterial depletion by 16S rRNA gene PCR amplification is presented in **Fig. 4b**.

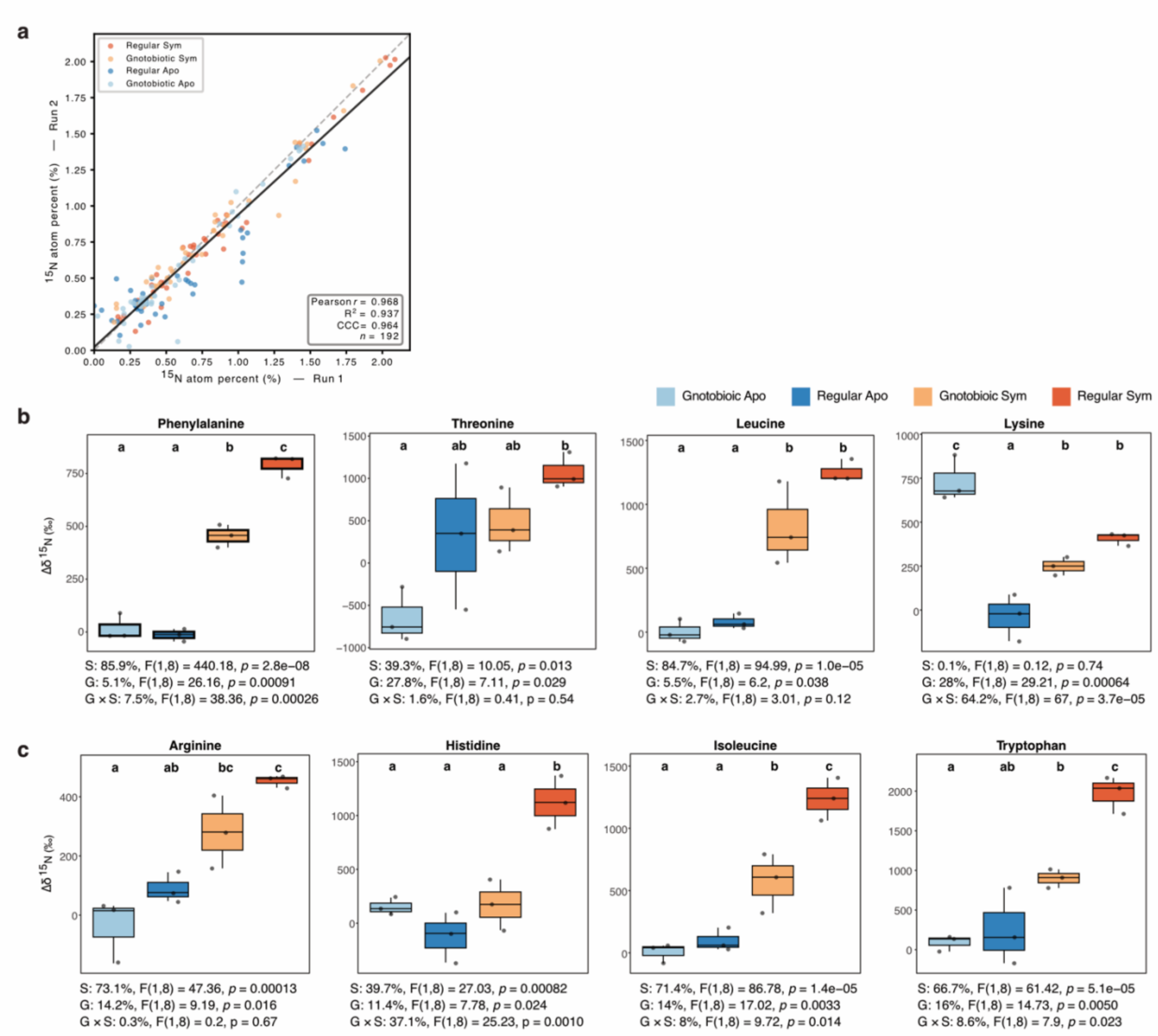

Supplementary Fig. 5: Technical replicate validation of UHPLC-HR-MS ^{15}N quantification.

a, Concordance between the primary (Run 1) and technical replicate (Run 2) injections, performed consecutively on the same analytical day. Each point represents one paired measurement of ^{15}N atom percent in an individual amino acid from one biological replicate (n = 192 paired measurements: 4 experimental conditions × 8 amino acids × 2 treatments × 3 biological replicates), colored by experimental condition. **b, c,** Independent re-analysis of the technical replicate (Run 2) data using the same statistical workflow as the primary analysis (**Fig. 4c, d**), shown for the four amino acids primarily influenced by algal

symbiosis (**b:** phenylalanine, threonine, leucine, lysine) and the four amino acids influenced by both bacterial and algal symbionts (**c:** arginine, histidine, isoleucine, tryptophan). Boxplots show ^{15}N enrichment ($\Delta\delta^{15}N$, ‰) across the four experimental conditions; center lines represent the median, bounds of boxes indicate the interquartile range, and individual data points represent biological replicates ($n = 3$ biological replicates per group). Statistical significance was assessed using two-way ANOVA with symbiotic state (S) and germ status (G) as factors; percentages indicate the relative variance contribution of each factor and their interaction ($S \times G$), with F-statistics and p-values reported below each panel. Letters above boxes denote significant pairwise differences based on Tukey's HSD post hoc tests ($p < 0.05$). The Run 2 results recapitulate all major group-level patterns and statistical interactions observed in the primary run (**Fig. 4c, d**), confirming the robustness of the ^{15}N enrichment conclusions to instrument-level technical variance. Source data are provided as a Source Data file.

References

1 Cui, G. *et al.* Molecular insights into the Darwin paradox of coral reefs from the sea anemone Aiptasia. *Sci. Adv.* **9**, eadf7108 (2023).
