# Supplementary material for: Algal symbiosis adaptively restructures the cnidarian microbiome to enhance holobiont metabolism
Source: Nat Commun. 2026 Nov 2;17:11128. doi: 10.1038/s41467-026-79238-6 (PMC13728110; doi:10.1038/s41467-026-79238-6)
Supplement: Supplementary file 2 [file 41467_2026_79238_MOESM2_ESM.pdf]

<u>Description Of Additional Supplementary Files</u>

Supplementary Data 1: Sequencing quality metrics

Supplementary Data 2: Family- and genus-level taxonomic profiles

Supplementary Data 3: Alpha and beta diversity statistics

Supplementary Data 4: Filtered normalized microbial transcript abundances

Supplementary Data 5: Pairwise microbial differential expression results

Supplementary Data 6: GO enrichment results for microbial DE genes

Supplementary Data 7: GSVA pathway activity scores

Supplementary Data 8: Limma statistics for GSVA pathway activity

Supplementary Data 9: Order-level bacterial abundance statistics

Supplementary Data 10: Bacteria, archaea, and virus abundance statistics

Supplementary Data 11: Phylum-level bacterial transcript abundance statistics

Supplementary Data 12: Primary-run 15N enrichment statistics

Supplementary Data 13: Technical-replicate 15N enrichment statistics
