# Supplementary material for: Algal symbiosis adaptively restructures the cnidarian microbiome to enhance holobiont metabolism
Source: Nat Commun. 2026 Nov 2;17:11128. doi: 10.1038/s41467-026-79238-6 (PMC13728110; doi:10.1038/s41467-026-79238-6)
Supplement: Supplementary file 3 [file 41467_2026_79238_MOESM4_ESM.pdf]

nature portfolio

Corresponding author(s): Guoxin Cui, Manuel Aranda

Last updated by author(s): Apr 17, 2026

Reporting Summary

Nature Portfolio wishes to improve the reproducibility of the work that we publish. This form provides structure for consistency and transparency in reporting. For further information on Nature Portfolio policies, see our Editorial Policies and the Editorial Policy Checklist.

Statistics

For all statistical analyses, confirm that the following items are present in the figure legend, table legend, main text, or Methods section.

n/a	Confirmed	
☐	☒	The exact sample size (*n*) for each experimental group/condition, given as a discrete number and unit of measurement
☐	☒	A statement on whether measurements were taken from distinct samples or whether the same sample was measured repeatedly
☐	☒	The statistical test(s) used AND whether they are one- or two-sided<br>*Only common tests should be described solely by name; describe more complex techniques in the Methods section.*
☐	☒	A description of all covariates tested
☐	☒	A description of any assumptions or corrections, such as tests of normality and adjustment for multiple comparisons
☐	☒	A full description of the statistical parameters including central tendency (e.g. means) or other basic estimates (e.g. regression coefficient) AND variation (e.g. standard deviation) or associated estimates of uncertainty (e.g. confidence intervals)
☐	☒	For null hypothesis testing, the test statistic (e.g. *F*, *t*, *r*) with confidence intervals, effect sizes, degrees of freedom and *P* value noted<br>*Give P values as exact values whenever suitable.*
☒	☐	For Bayesian analysis, information on the choice of priors and Markov chain Monte Carlo settings
☒	☐	For hierarchical and complex designs, identification of the appropriate level for tests and full reporting of outcomes
☐	☒	Estimates of effect sizes (e.g. Cohen's *d*, Pearson's *r*), indicating how they were calculated

Our web collection on statistics for biologists contains articles on many of the points above.

Software and code

Policy information about availability of computer code

Data collection	Laser microdissection software (Leica LMD 6000) was used for tissue microdissection. High-throughput sequencing data were collected using an Illumina HiSeq 2000 system. Metabolomics data acquisition was conducted using Xcalibur v1.6 software on a Dionex Ultimate 3000 UHPLC system coupled with a Q Exactive Plus mass spectrometer (Thermo Fisher Scientific).
Data analysis	Read quality trimming and adapter removal were performed using Trimmomatic (v0.32). Sequence error correction was conducted with SPAdes (v3.9.0), and ribosomal RNA reads were partitioned using SortMeRNA (v2.1b). Assembled 16S rRNA gene transcripts were processed using PANDAseq (v2.11) and taxonomically classified via MOTHUR (v1.39.5) with the GreenGenes database (v13_8). Protein-coding transcripts were assembled de novo using Trinity (v2.2.0), and coding sequences were predicted using TransDecoder (v2.0.1). Gene catalog clustering was performed using CD-HIT (v4.8.1), and functional/taxonomic annotations were assigned using the KMAP platform referencing the KEGG Orthology database. Complementary taxonomy profiling was executed via MetaPro (v3.0.1) for mRNA and Kraken2 (v2.1.3) with the PlusPF database for rRNA. Transcript quantification was calculated using kallisto (v0.43.0) and differential gene expression was evaluated with sleuth (v0.27.0). Differential pathway and functional enrichment analyses were carried out using topGO (v2.52.0), GSVA (v1.48.0 or equivalent), and limma (v3.60.3). ComplexHeatmap (v2.21.2) and the base stats/vegan packages in R (v4.3.2) were used for data visualization, alpha/beta diversity, and statistical analyses (two-way ANOVA, Tukey's HSD, PERMANOVA, PERMDISP, t-tests). Target metabolomics data processing and isotopic signal quantification were performed in Xcalibur v1.6 (Thermo Fisher Scientific).<br>The complete analytical pipeline and source code available at GitHub: https://github.com/1cuigx/tissue-specific-microbiome. And the repository has been archived in Zenodo and is available at https://doi.org/10.5281/zenodo.22806098

For manuscripts utilizing custom algorithms or software that are central to the research but not yet described in published literature, software must be made available to editors and reviewers. We strongly encourage code deposition in a community repository (e.g. GitHub). See the Nature Portfolio guidelines for submitting code & software for further information.

Data

Policy information about availability of data

All manuscripts must include a data availability statement. This statement should provide the following information, where applicable:

- Accession codes, unique identifiers, or web links for publicly available datasets
- A description of any restrictions on data availability
- For clinical datasets or third party data, please ensure that the statement adheres to our policy

Raw data generated from UHPLC-HR-MS analysis have been deposited in the NIH Common Fund's National Metabolomics Data Repository under Project ID PR002710 [http://dx.doi.org/10.21228/M8C25W]. RNA-seq data generated in this study have been deposited in the NCBI Sequence Read Archive under BioProject accession code PRJNA631577 [https://www.ncbi.nlm.nih.gov/bioproject/PRJNA631577/]. The analysis code and input data required to reproduce the main and supplementary display items are provided in the associated GitHub repository [https://github.com/1cuigx/tissue-specific-microbiome], and archieved at Zenodo [https://doi.org/10.5281/zenodo.22806098]. All other relevant data are provided in the Supplementary Data. Source data are provided with this paper.

Research involving human participants, their data, or biological material

Policy information about studies with human participants or human data. See also policy information about sex, gender (identity/presentation), and sexual orientation and race, ethnicity and racism.

Reporting on sex and gender	Not applicable.
Reporting on race, ethnicity, or other socially relevant groupings	Not applicable.
Population characteristics	Not applicable.
Recruitment	Not applicable.
Ethics oversight	Not applicable.

Note that full information on the approval of the study protocol must also be provided in the manuscript.

Field-specific reporting

Please select the one below that is the best fit for your research. If you are not sure, read the appropriate sections before making your selection.

☒ Life sciences ☐ Behavioural & social sciences ☐ Ecological, evolutionary & environmental sciences

For a reference copy of the document with all sections, see nature.com/documents/nr-reporting-summary-flat.pdf

Life sciences study design

All studies must disclose on these points even when the disclosure is negative.

Sample size	Sample sizes were determined empirically based on prior experience with the experimental systems and established practice for the respective experimental approaches. Four biological replicates were used for sequencing experiments and three biological replicates were used for metabolomics experiments. No formal a priori power calculation was performed. These sample sizes were chosen to provide independent biological replication and to capture biological variation while enabling reproducible detection of differences between experimental groups.
Data exclusions	No data was excluded from our analysis.
Replication	Biological replicates were used for all experiments, and all attempts at replication were successful.
Randomization	The allocation of samples in each parallel experiment was randomly applied.
Blinding	Blinding was not applicable to most experiments because the experimental procedures and measurements did not involve subjective assessment or investigator-dependent outcome scoring.

Reporting for specific materials, systems and methods

We require information from authors about some types of materials, experimental systems and methods used in many studies. Here, indicate whether each material, system or method listed is relevant to your study. If you are not sure if a list item applies to your research, read the appropriate section before selecting a response.

Materials & experimental systems

n/a	Involved in the study
☒	☐ Antibodies
☒	☐ Eukaryotic cell lines
☒	☐ Palaeontology and archaeology
☐	☒ Animals and other organisms
☒	☐ Clinical data
☒	☐ Dual use research of concern
☒	☐ Plants

Methods

n/a	Involved in the study
☒	☐ ChIP-seq
☒	☐ Flow cytometry
☒	☐ MRI-based neuroimaging

Animals and other research organisms

Policy information about studies involving animals; ARRIVE guidelines recommended for reporting animal research, and Sex and Gender in Research

Laboratory animals	Sea anemone Exaiptasia diaphana CC7 strain
Wild animals	No wild animals were involved.
Reporting on sex	Sex was not determined. Aiptasia used in this study were maintained as laboratory cultures, and sex was not a variable examined in the study.
Field-collected samples	No field-collected samples were involved.
Ethics oversight	Ethics oversight was not applicable. The study used the sea anemone Aiptasia, an invertebrate that is not subject to institutional animal research regulations applicable to vertebrate animals.

Note that full information on the approval of the study protocol must also be provided in the manuscript.

Plants

Seed stocks	No plant was involved in this study.
Novel plant genotypes	Not applicable.
Authentication	Not applicable.
