# Supplementary material for: Algal symbiosis adaptively restructures the cnidarian microbiome to enhance holobiont metabolism
Source: Nat Commun. 2026 Nov 2;17:11128. doi: 10.1038/s41467-026-79238-6 (PMC13728110; doi:10.1038/s41467-026-79238-6)
Supplement: Supplementary file 4 [file 41467_2026_79238_MOESM5_ESM.pdf]

nature portfolio

Peer Review File

Algal symbiosis adaptively restructures the cnidarian microbiome to enhance holobiont metabolism

Corresponding Author: Professor Guoxin Cui

This file contains all reviewer reports in order by version, followed by all author rebuttals in order by version.

Version 0:

Reviewer comments:

Reviewer #1

(Remarks to the Author)
Thank you for the opportunity to review the manuscript by Cui and colleagues entitled, “Algal symbiosis adaptively restructures the cnidarian microbiome to enhance holobiont metabolism” for consideration for publication in Nature Communications.

This study used tissue-resolved laser-microdissection of Aiptasia anemones, metatranscriptomics, and 15N nitrogen tracing experiments to show spatially compartmentalized microbial communities and metabolism that enhance holobiont nitrogen assimilation and functioning. The study is innovative and well-executed and expands our understanding of the interactive roles of the symbiotic relationship and the microbiome in contributing to holobiont function and nutritional cycling. The authors expand this work beyond description of the microbial community by linking microbial nitrogen metabolism to host amino acid synthesis using a combination of approaches. The spatially resolved integration of microbial and host nitrogen metabolism is conceptually novel and represents an important mechanistic step beyond descriptive cnidarian microbiome studies. These results will be of wide interest and the integration of metatranscriptomics, and stable isotope tracing is compelling. Therefore, I believe that this manuscript is a strong candidate for publication in Nature Communication because it is rigorous, conceptually integrative, and relevant to symbiotic systems.

However, there are several areas of this manuscript that require revision prior to publication. These include adding missing details to the methods section, providing statistical evidence for claims in the results section, increasing data availability, and strengthening discussion of limitations and caveats of the approaches used in this study. I have detailed these recommendations below.

MAJOR COMMENTS
1. The methods section is missing key methodological details required to interpret the results and to replicate the study. First, the authors must explicitly state the sample size (biological replicates) for all data sets and analyses performed. Here are some example statements where sample size is required (note that this list is not exhaustive and the authors should confirm sample size is thoroughly reported):
- Line 104: How many total animals were sampled? Were these sampled from the 8 tanks mentioned above for RNA-seq experiments? Please state total sample size.
- Line 112-113: How many regions of interest were dissected per animal of each tissue type?
- Line 166: What was the sample size of apo and sym anemones for gnotobiotic generation?
- Lines 176-178: What is the sample size for isotope labeling experiments?

2. In addition, the methods section is missing critical information on statistical methods for most analyses. Please add the necessary details for the reader to access information including statistical tests performed, model structure, checks for assumptions, transformations (if used), batch effect handling (if relevant), multiple-testing controls, and post hoc procedures for all tests and data sets. This may allow authors to streamline the results section by moving methods descriptions to the methods (e.g., lines 305-307, lines 347-349). Here are several examples of missing statistical information in the methods section (again this list is not exhaustive but is meant to point out examples):
- In the caption of Fig 1, the authors state that ANOVA and variance portioning tests were used, but these are not described in the methods section.
- In lines 295-296, authors state they used a PCA, but this was also not described in the methods. Please add statistical

methods for all tests and comparisons in the methods section.
- The tests described in the caption of Fig 4 that should be detailed in the methods section.
- Lines 153-155: What groups were compared to generate “differentially expressed” genes? Please specify the model structure used to identify DEGs.
- Line 361: Multiple t-tests appear to be performed (for each compound). Please specify how you corrected for multiple comparisons.

3. Provide context and rationale for the concentration and duration of the 15N tracer experiments and whether nitrogen monitoring was performed throughout the two-week experiment. Further, please add the light conditions used throughout the experiment.

4. The gnotobiotic experiment appears to have relied only on culture to verify the presence of microbes following gnotobiotic treatment. However, this method does not account for non-culturable bacteria, which may have been present. Please add information in the manuscript for how you verified that non-culturable bacteria were not present (if conducted, e.g., molecular methods). If this was not done, the authors must add statements in the discussion section that address the possibility that other bacteria may have been present but were not detected by the culture method.

5. The results statement is largely missing statistical results and justification for statements. Please add statistical results to all results statements in the manuscript. For example, in lines 253-261 and lines 268-271, the authors state which taxa were significantly enriched. However, these statements do not include statistical results or reference supporting tables with results. Please add the relevant test results (e.g., SS, DF, F, and p values) for each statement and the name of the test performed. Additional examples include lines 283-284, 285-286, 303-305, and 366-378. These statistical results are included in part in the figures but should be reported fully in the text. If the authors would like to cite only the p-value in the text, please provide and reference supplementary tables with full statistical results. This is essential to substantiate the statements in the text, particularly those regarding the “synergy” of microbial and symbiont communities. This would require significant interactive effects in statistical tests between bacteria and algal symbiont presence (e.g., lines 369-371) and these results should be explicitly stated.

6. The manuscript would be improved by addition of statements in the introduction and/or methods section that roadmap the objectives and tests performed along with hypotheses. For example, lines 333-349 in the results provide welcome context for the purpose of gnotobiotic comparisons that would assist the reader in understanding the purpose of these tests earlier in the manuscript. As someone who is not an expert with these methods, I found the roadmap statements helpful. I recommend the authors add brief statements in the introduction and/or methods section that outline the hypothesis tested and purpose for each experiment/test performed.

7. Data availability: Please provide a link to online repositories that contain the code and data used for all statistical analyses presented in the manuscript. Please update the accession numbers with active accession numbers prior to publication.

8. Finally, the discussion would benefit from a short section acknowledging limitations, including reliance on culturing to define gnotobiotic conditions and the transferability of results from Aiptasia to reef-building corals.

MINOR COMMENTS

Methods Section
- Lines 90-92: Sample size of the “subset of aposymbiotic anemones inoculated with…”. Also in this section, how was a “stable symbiont population” measured? Were all anemones in the same tank or different tanks during the culture period?
- Line 128: What was your threshold for removing “low-quality bases”? Please add any additional thresholds or filtering parameters used in RNA-seq data analysis.
- Line 135-136: Please list the specific options and parameters used in the MOTHUR pipeline for taxonomic classification and the version of the GreenGenes database used in analysis. However, GreenGenes is a smaller database and is not updated as frequently as SILVA. The authors should consider performing the analysis to the most recent SILVA database, or alternatively, provide justification for why GreenGenes was used.
- Line 222-223: Please describe the statistical methods used for “comparative analyses”.
Results Section
- The authors highlight 4 categories of functional enrichment terms in the results in Fig 3. Please provide 1-2 sentences in which you describe why these 4 were chosen specifically.
- There are several statements in the results that are interpretive and are better suited for the discussion (e.g., lines 327-330).

Overall, I find this to be a rigorous and well-designed study with important implications for understanding holobiont nitrogen cycling. Pending addition of key methodological details, statistical transparency, and expanded discussion of limitations, it will make a valuable contribution suitable for publication in Nature Communications.

Reviewer #2

(Remarks to the Author)
Overall, this is a well-written and well-executed set of experiments. The data, I believe, could be explored further to yield a more impactful story and more actionable insights. I think it is worthy of publication, and my recommendation is minor revision. While I am recommending some data re-analysis, I do not think my suggestions go far beyond what the authors may have already done. I find it strange that there is no supplemental information included with this manuscript. Several of my suggested points may be better suited for a supplement, depending on their impact on the overall story. Nevertheless, I see these points as integral to presenting a comprehensive set of results for the reader.

What are the noteworthy results?

The paper describes a carefully controlled experiment with novel and informative results concerning the distribution of microbial species across cnidarian tissues in the presence and absence of algal symbionts. The results indicate shifts in the anemone's microbiome dependent on symbiotic state. The paper is unique in examining these trends in a tightly controlled and tissue-specific manner using laser microdissection.

In the introduction, the authors describe many microbiome studies as failing to explore functional implications in depth. While I appreciate the dual transcriptomic–metabolomic approach used here, I do not find the results to dig much deeper than other papers. KOG enrichments are standard for RNA-seq analysis, and although tissue-specific conditions are rare in cnidarian studies, the authors could extract more information and value from this dataset.

- Will the work be of significance to the field and related fields? How does it compare to the established literature? -

I find this work to be significant and to contribute to the field. A stronger and deeper understanding of cnidarian–algal symbiosis is essential for coral-bleaching mitigation and for investigating the evolution of endosymbiosis more generally. The paper adds important knowledge to the literature. In particular, it shows that:

1. nitrite assimilation pathways are favored in symbiosis;
2. certain essential amino acids are primarily produced by the symbionts, while levels of others are enhanced by particular microbes;
3. microbial communities in cnidarians exhibit tissue-specific compartmentalization, and these associations may be metabolically driven.

However, although I find the approach and methods impactful, the results presented are not, in my opinion, as impactful as they could be if the data were explored in greater detail. I offer some suggestions below that I think should be strongly considered for acceptance.

- Does the work support the conclusions and claims? Are there flaws in the data analysis or interpretation? -

The work does support the current conclusions. However, I am somewhat disappointed in the depth of analysis presented. This appears to be a potentially very rich dataset, yet the analyses are, in my opinion, somewhat surface-level. Below are several comments and suggestions for additional analyses or clarifications that could make this a more impactful contribution.

1. Unannotated gene cluster in Figure 3F:
There is a cluster of genes at the top of the heat map that appears to have the strongest signal and interaction for gastrodermis and symbiont. There is no annotation of the potential functions in that cluster. Perhaps none were found, or perhaps the authors considered them irrelevant to nitrogen cycling, but they should be discussed. If no annotations were obtained, I suggest further BLAST searches against both microbial and eukaryotic databases; some sequences may be protist or anemone genes that slipped through filtering.

2. Suspicious band in Figure 3E:
In the SymGas/ApoGas comparison, there is a strange band showing nearly no genes with ~2-fold change. That seems suspicious and suggests something may be off with the fold-change analysis for that comparison. The authors should investigate this further.

3. Lack of anemone transcriptomic discussion:
The absence of analysis of anemone transcripts is surprising. Perhaps this is intended for another paper, but presenting the story holistically would be far more impactful. At minimum, I would like to know whether any complementary pathways are differentially regulated in the anemones—e.g., vitamin biosynthesis or metabolic pathways that might interact with changes in microbes or symbionts.

4. Limited taxonomic resolution:
The text mentions B. subtilis and Klebsiella, but there is no broader taxonomic breakdown. Why is the data presented only at a high taxonomic level? If read quality or abundance prevents more precise taxonomic assignment, this should be clearly stated rather than left implicit.

5. Lack of RNA data and microbial confirmation in gnotobiotic treatments:
Was there really no RNA-seq data for the gnotobiotic treatments? This would have enabled fascinating comparisons to separate symbiosis-specific changes from microbial influences. I would also have liked to see 16S abundance data from the

gnotobiotic samples to confirm axenity. A single low-resolution photo of petri dishes is not convincing.

6. Lack of visuals for laser microdissection:
I am surprised that there are no visuals of the laser microdissection process. Some should be included in the supplement.

7. Insufficient statistics on sequencing quality:
I would also recommend including metrics on read quality, read depth, etc., by sample. as these are important in understanding the limitations of the conclusions that can be drawn from the data.

- Is the methodology sound? Is there enough detail for reproducibility? -

The methods and execution of the experiment are largely sound. However, a few points are unclear and would benefit from clarification for reproducibility and for interpretive context:

1. Brine shrimp sterility:
Were the brine shrimp also grown and hatched in sterile seawater? If so, how often and by what methods was the hatchery disinfected or sterilized between cultures?

2. Feeding of gnotobiotic anemones:
Were the gnotobiotic anemones fed? This is important for reproducibility and for interpreting the metabolomics data. If they were not fed during/after antibiotic exposure, when were they last fed, and was this consistent across replicates and conditions?

3. Technical replicates in metabolomics:
Were any technical replicates performed for the metabolite analysis? From my reading, it seems not. If samples are still preserved, I suggest re-running with at least two technical replicates.

Reviewer #3

(Remarks to the Author)
The manuscript addresses an important and timely question in cnidarian holobiont biology and provides evidence that symbiogenesis with photosynthetic dinoflagellates drives a tissue-specific reorganisation of Aiptasia microbiome, with functional consequences for nitrogen assimilation at the holobiont level. These findings advance the field by linking spatial microbiome structure to holobiont metabolic function. The integration of tissue-resolved metatranscriptomics, gnotobiology and δ15N analyses is innovative and the study has the potential to make a significant impact. The data generally support the conclusions, and the methodology is sound; however, the manuscript currently lacks sufficient detail for reproducibility regarding statistical and isotopic analyses, and several figures require clarification. Addressing these issues will strengthen the manuscript and ensure it meets the standards expected for Nature Communications.
A dedicated and comprehensive Statistical Analysis section must be added to the Methods, following Nature Communications guidelines. Without this section, the robustness of the analyses cannot be evaluated. The manuscript appears not to include any Supplementary Information. If space becomes limiting when the authors add a proper statistical analysis section, some content could be moved to the Supplementary Materials.
Figure 1:
Figure 1a: Archaea are absent from the bars. Please clarify whether they were not detected, filtered out, or omitted unintentionally.
Please report sample sizes for each group in boxplots.
Figure 1b: The manuscript only describes most abundant bacterial groups, but should also discuss differences among the four tissue types compared that are not obvious from the boxplots. Please also comment on within-group variability.
Although it is explained in the Results section, it remains rather confusing that the bacterial groups shown in Fig. 1b do not correspond to those represented in the boxplots. In the boxplot panels, it would be helpful to indicate clearly which of the groups displayed in Fig. 1b each boxplot refers to. In addition, I suggest using a different colour palette for the boxplots to avoid confusion with Fig. 1a and 1b. Post-hoc analyses after significant ANOVAs have to be performed and significant differences shown in figures. In cases where significant tissue × symbiosis interactions are detected, it would be helpful to elaborate on their biological relevance and interpret these effects more explicitly.
The same comments apply to other figures, including sample sizes and missing post-hoc results.
In line 270, you claim that bacteria and archaea are significantly enriched in the symbiotic gastrodermis (Fig. 2a,b); this requires supporting post-hoc statistics. Viral abundances may not differ significantly between tissues. Please evaluate and report accordingly.
Figure 3:
The interpretation of microbial DEGs in line 296 can be further supported by group separation in the PCA plots. I suggest explicitly mentioning that PC1 explains >50% of the variance in Fig. 3b and 3d, compared to ~27% in 3a and 3c.
Have the authors attempted a single PCA including all four groups simultaneously? If so, do the conclusions remain consistent?
Explanation of Fig. 3e in the figure caption requires improvement, including an explanation of why (only) expression of genes of nitrite reductase is shown. Which statistical test was performed?
Lines 323–324 report p-values without specifying the test used. This must be corrected following the journal statistical-reporting guidelines. In general, I suggest the authors to improve statistical reporting in figures and within results according

to the journal standards.
In Fig. 3f, please explain why some groups show high apparent microbial gene activity (intense red, on the top of the figure) but no specific pathways are indicated in those cases.
I am aware that compound-specific stable isotope analyses of amino acids are costly. Nevertheless, assessing the carbon sources of essential amino acids would have been valuable for more directly linking cnidarian nutrition with the contributions of microbial symbionts.
Figure 4:
In Figures 4b, 4c, and 4d, the values represent 15N enrichment relative to control samples. To clarify that the figure shows a difference from the control, rather than an absolute tissue value, it would be more accurate to label the axes as "15N enrichment" or "Δδ15N". It may also be worth noting in the results that in aposymbiotic organisms, several amino acids (Phe, Thr, Leu, Lys in Fig. 4b) display lower values relative to controls, an effect not observed for the amino acids shown in Fig. 4c. For Figure 4, the same comments made for Figure 1 apply. Please indicate sample sizes clearly and report the results of the post-hoc tests.

Version 1:

Reviewer comments:

Reviewer #2

(Remarks to the Author)
Thank you for taking the time to complete such thorough revisions and updates to the paper. I think the findings now offer a much more holistic and informative picture to the reader than in the previous version. I am glad to see that the additional analyses and methods further strengthened the original conclusions and did not uncover any technical or analytical artifacts. I feel satisfied that all my reviewer comments were adequately addressed and believe the paper is recommendable for publication.

Reviewer #3

(Remarks to the Author)
The authors have adequately addressed my comments, and I am pleased with the revisions made to the manuscript.

Open Access This Peer Review File is licensed under a Creative Commons Attribution 4.0 International License, which permits use, sharing, adaptation, distribution and reproduction in any medium or format, as long as you give appropriate credit to the original author(s) and the source, provide a link to the Creative Commons license, and indicate if changes were made.
In cases where reviewers are anonymous, credit should be given to 'Anonymous Referee' and the source.
The images or other third party material in this Peer Review File are included in the article's Creative Commons license, unless indicated otherwise in a credit line to the material. If material is not included in the article's Creative Commons license and your intended use is not permitted by statutory regulation or exceeds the permitted use, you will need to obtain permission directly from the copyright holder.
To view a copy of this license, visit https://creativecommons.org/licenses/by/4.0/

REVIEWER COMMENTS

Reviewer #1 (Remarks to the Author):

Thank you for the opportunity to review the manuscript by Cui and colleagues entitled, “Algal symbiosis adaptively restructures the cnidarian microbiome to enhance holobiont metabolism” for consideration for publication in Nature Communications.

This study used tissue-resolved laser-microdissection of Aiptasia anemones, metatranscriptomics, and 15N nitrogen tracing experiments to show spatially compartmentalized microbial communities and metabolism that enhance holobiont nitrogen assimilation and functioning. The study is innovative and well-executed and expands our understanding of the interactive roles of the symbiotic relationship and the microbiome in contributing to holobiont function and nutritional cycling. The authors expand this work beyond description of the microbial community by linking microbial nitrogen metabolism to host amino acid synthesis using a combination of approaches. The spatially resolved integration of microbial and host nitrogen metabolism is conceptually novel and represents an important mechanistic step beyond descriptive cnidarian microbiome studies. These results will be of wide interest and the integration of metatranscriptomics, and stable isotope tracing is compelling. Therefore, I believe that this manuscript is a strong candidate for publication in Nature Communication because it is rigorous, conceptually integrative, and relevant to symbiotic systems.

However, there are several areas of this manuscript that require revision prior to publication. These include adding missing details to the methods section, providing statistical evidence for claims in the results section, increasing data availability, and strengthening discussion of limitations and caveats of the approaches used in this study. I have detailed these recommendations below.

We thank the reviewer for the careful and constructive evaluation of our manuscript, and for the specific, actionable recommendations. We have revised the manuscript to address each point, with particular attention to expanded statistical reporting in the Results, a new Statistical Analysis subsection in the Methods, molecular validation of the gnotobiotic state, and a dedicated limitations section in the Discussion. Our point-by-point responses to each specific comment are provided below.

MAJOR COMMENTS

1. The methods section is missing key methodological details required to interpret the results and to replicate the study. First, the authors must explicitly state the sample size (biological replicates) for all data sets and analyses performed. Here are some example statements where sample size is

required (note that this list is not exhaustive and the authors should confirm sample size is thoroughly reported):

- Line 104: How many total animals were sampled? Were these sampled from the 8 tanks mentioned above for RNA-seq experiments? Please state total sample size.

- Line 112-113: How many regions of interest were dissected per animal of each tissue type?

- Line 166: What was the sample size of apo and sym anemones for gnotobiotic generation?

- Lines 176-178: What is the sample size for isotope labeling experiments?

We thank the reviewer for highlighting these omissions. We have audited the Methods and added the exact sample sizes (biological replicates, *n*) and procedural details to all relevant text sections. Specifically:

- **RNA-seq sampling** (Lines 455–456): we clarified that 8 total anemones (*n* = 4 symbiotic, *n* = 4 aposymbiotic) were sampled from 8 independent experimental tanks.
- **Laser microdissection** (Lines 465–468): we specified the targeted tissue area (~2–3 mm2 per tissue type) and provided the estimated number of ROIs dissected to achieve this area (30–50 for the gastrodermis and 40–80 for the epidermis).
- **Gnotobiotic animal generation** (Lines 579–583; 594–599): we expanded this section to describe the batch setup (four beakers of ~30 anemones) and the validation design (*n* = 2 anemones per beaker each for culture-based and 16S rRNA PCR validation). Full details and the PCR gel image (Fig. 4b) are addressed in our response to Comment 4 below.
- **Isotope labeling** (Lines 606–608): we now outline the experimental design, confirming n = 3 biological replicates for each of the 8 conditions, yielding a total of 24 anemones.

2. In addition, the methods section is missing critical information on statistical methods for most analyses. Please add the necessary details for the reader to access information including statistical tests performed, model structure, checks for assumptions, transformations (if used), batch effect handling (if relevant), multiple-testing controls, and post hoc procedures for all tests and data sets. This may allow authors to streamline the results section by moving methods descriptions to the methods (e.g., lines 305-307, lines 347-349). Here are several examples of missing statistical information in the methods section (again this list is not exhaustive but is meant to point out examples):

- In the caption of Fig 1, the authors state that ANOVA and variance portioning tests were used, but these are not described in the methods section.

- In lines 295-296, authors state they used a PCA, but this was also not described in the methods. Please add statistical methods for all tests and comparisons in the methods section.

- The tests described in the caption of Fig 4 that should be detailed in the methods section.

- Lines 153-155: What groups were compared to generate "differentially expressed" genes? Please specify the model structure used to identify DEGs.

- Line 361: Multiple t-tests appear to be performed (for each compound). Please specify how you corrected for multiple comparisons.

We thank the reviewer for this constructive feedback. To streamline the text as suggested, we have created a dedicated **Statistical Analysis** section at the end of the Methods. Furthermore, the full outputs of all statistical models are now provided as **Supplementary Data**.

To address your specific examples:

- Fig 1 and Fig 4 tests: the two-way ANOVAs, variance partitioning (sum of squares), and post hoc procedures (Tukey's HSD) are now explicitly detailed in the Statistical Analysis section (Lines 666–681).
- PCA: the PCA methodology and data transformation step (log2(TPM + 1)) have been added to the Differential expression and functional analysis section (Lines 543–550; 557–561).
- DEG model: we have expanded the metatranscriptome analysis section to explicitly state the single-factor model structure (~ condition) and the Wald tests for the four pairwise comparisons (Lines 551–557).
- Multiple testing: We have clarified in the Methods that all *p*-values across multiple taxa and metabolites were adjusted using the Benjamini-Hochberg FDR procedure (Lines 556–557; 678–681).

3. Provide context and rationale for the concentration and duration of the 15N tracer experiments and whether nitrogen monitoring was performed throughout the two-week experiment. Further, please add the light conditions used throughout the experiment.

We thank the reviewer for requesting these important experimental clarifications. We have updated the Methods (Lines 601–612) to explicitly detail the rationale for our experimental design and the environmental conditions during the incubation.

Specifically, we used a 30 μM ^{15}N-nitrite concentration. While higher than ambient reef levels, this concentration is comparable to those used in previous laboratory stable isotope tracing studies (e.g., Burris 1984, Pernice et al., 2012, Kopp et al., 2013). It was chosen to provide a robust tracer signal strong enough to detect incorporation into downstream metabolites (such as individual amino acids) over the incubation period, without inducing toxicity. A two-week incubation was

selected to allow sufficient time for *de novo* amino acid synthesis and subsequent translocation across holobiont tissues, which are expected to turn over more slowly than the initial uptake of inorganic nitrogen.

Regarding the environmental conditions, the anemones were maintained consistently across all experiments under a 12 h:12 h light:dark cycle (~40 µmol photons m^2 s^{-1}). To prevent ^{14}N-rich food from diluting the ^{15}N signal, the anemones were starved throughout the two-week incubation. We did not continuously monitor nitrogen concentrations; the 30 µM tracer concentration was maintained by complete water and treatment changes every 48 hours.

4. The gnotobiotic experiment appears to have relied only on culture to verify the presence of microbes following gnotobiotic treatment. However, this method does not account for non-culturable bacteria, which may have been present. Please add information in the manuscript for how you verified that non-culturable bacteria were not present (if conducted, e.g., molecular methods). If this was not done, the authors must add statements in the discussion section that address the possibility that other bacteria may have been present but were not detected by the culture method.

As noted in our response to Comment 1, we agree with the reviewer that culture-based methods alone are insufficient to rule out unculturable bacteria. Our original experimental protocol already included a parallel molecular validation (16S rRNA PCR with primers 67F/1492R), which we performed alongside the plating but inadvertently omitted from the first submission. We have now updated the Methods section to describe both the culture-based validation and molecular validation of the gnotobiotic state, and added the PCR gel results as Fig. 4b alongside the plating images (Fig. 4a; Supplementary Fig. 4).

5. The results statement is largely missing statistical results and justification for statements. Please add statistical results to all results statements in the manuscript. For example, in lines 253-261 and lines 268-271, the authors state which taxa were significantly enriched. However, these statements do not include statistical results or reference supporting tables with results. Please add the relevant test results (e.g., SS, DF, F, and p values) for each statement and the name of the test performed. Additional examples include lines 283-284, 285-286, 303-305, and 366-378. These statistical results are included in part in the figures but should be reported fully in the text. If the authors would like to cite only the p-value in the text, please provide and reference supplementary tables with full statistical results. This is essential to substantiate the statements in the text, particularly those regarding the "synergy" of microbial and symbiont communities. This would require significant interactive effects in statistical tests between bacteria and algal symbiont presence (e.g., lines 369-371) and these results should be explicitly stated.

We thank the reviewer for this constructive feedback. We have revised the Results section throughout and report statistical tests and adjusted p-values for every quantitative claim. As the reviewer suggested, the main text now cites the test performed and the adjusted p-values directly, while full model outputs, including SS, DF, F-values, and variance partitioning, are provided in Supplementary Data 9–13.

On the specific point about "synergy", we now report interaction terms from the two-way ANOVAs for the four amino acids showing synergistic responses directly in the Results (lines 312–313). These significant interaction effects (adjusted $p < 0.05$) support the synergistic enhancement of holobiont nitrogen assimilation described in the text, with the full statistical outputs in Supplementary Data 12.

6. The manuscript would be improved by addition of statements in the introduction and/or methods section that roadmap the objectives and tests performed along with hypotheses. For example, lines 333-349 in the results provide welcome context for the purpose of gnotobiotic comparisons that would assist the reader in understanding the purpose of these tests earlier in the manuscript. As someone who is not an expert with these methods, I found the roadmap statements helpful. I recommend the authors add brief statements in the introduction and/or methods section that outline the hypothesis tested and purpose for each experiment/test performed.

We agree, and have added a roadmap paragraph at the end of the Introduction that outlines our hypotheses and the rationale for each experimental approach (Lines 66–80). This new paragraph first states our hypothesis that the bacterial community is compartmentalized between tissue layers and dynamically regulated by the algal symbiont. It then explains the logic of each approach: 16S rRNA profiling for spatial composition, metatranscriptomics for active functional shifts and metabolic outputs, and ^{15}N stable isotope probing with gnotobiotic and regular anemones to decouple bacterial from algal contributions.

7. Data availability: Please provide a link to online repositories that contain the code and data used for all statistical analyses presented in the manuscript. Please update the accession numbers with active accession numbers prior to publication.

We have now fully updated the Data Availability and Code Availability sections (Lines 693–703). All custom code and processed datasets used for the statistical analyses have been deposited at https://github.com/1cuigx/tissue-specific-microbiome. Raw sequencing data are deposited at

NCBI Sequence Read Archive (PRJNA631577), and metabolomic data are deposited at the National Metabolomics Data Repository (ST004292).

8. Finally, the discussion would benefit from a short section acknowledging limitations, including reliance on culturing to define gnotobiotic conditions and the transferability of results from Aiptasia to reef-building corals.

We thank the reviewer for this constructive suggestion. To address this, we have added a dedicated section limitations and future directions at the end of the Discussion (Lines 392–427), covering both points the reviewer raised. Regarding the gnotobiotic state, we acknowledge that even our combined culture-dependent and 16S rRNA PCR validation cannot entirely rule out trace residual or low-abundance unculturable taxa. For transferability, we note that Aiptasia lacks the calcium carbonate skeleton of scleractinian corals and therefore lacks the endolithic microenvironment or the distinct energetic demands associated with calcification.

MINOR COMMENTS

Methods Section

- Lines 90-92: Sample size of the "subset of aposymbiotic anemones inoculated with…". Also in this section, how was a "stable symbiont population" measured? Were all anemones in the same tank or different tanks during the culture period?

We thank the reviewer for bring our attention to this potential ambiguity. We have revised the Methods section (Lines 437–444) to clarify that this specific text describes the historical generation of our long-term CC7-SSB01 symbiotic and aposymbiotic laboratory stocks, rather than a specific experimental cohort. All anemones used in the experiments reported here were subsequently sourced from these established lines.

Regarding the question about tank allocations, we have added text explicitly stating that these stock anemones were communally cultured in polycarbonate tanks, while experimental tanks were set up according to each specific experimental design. Furthermore, we clarified that a stable symbiont population was ensured by culturing the anemones for approximately 8 weeks prior to any experimental use. Because Aiptasia typically reach symbiont carrying capacity within 4 weeks of inoculation, our 8-week timeline, combined with visual confirmation of uniform tissue browning, conservatively guarantees stability.

- Line 128: What was your threshold for removing “low-quality bases”? Please add any additional thresholds or filtering parameters used in RNA-seq data analysis.

We thank the reviewer for pointing out this omission. We have updated the Methods section to specify the parameters used in this process (Lines 486–490).

- Line 135-136: Please list the specific options and parameters used in the MOTHUR pipeline for taxonomic classification and the version of the GreenGenes database used in analysis. However, GreenGenes is a smaller database and is not updated as frequently as SILVA. The authors should consider performing the analysis to the most recent SILVA database, or alternatively, provide justification for why GreenGenes was used.

We have updated the Methods to include the Wang classifier and the 80% bootstrap threshold used in our MOTHUR pipeline, and confirmed our use of GreenGenes v13_8. We chose to retain the GreenGenes classification because our study focuses on broad, high-level community shifts rather than on fine-scale taxonomic identities. GreenGenes and SILVA are highly concordant at these ranks (e.g., Order, Phylum). Furthermore, using GreenGenes ensures direct comparability with foundational Aiptasia microbiome literature, while our core functional conclusions are independently supported by our metatranscriptomic and ^{15}N stable isotope data.

- Line 222-223: Please describe the statistical methods used for “comparative analyses”.

We have replaced "comparative analyses" with a detailed description of the statistical tests, now included in a dedicated Statistical Analysis subsection at the end of the Methods. Individual ^{15}N enrichment levels were analyzed using two-way ANOVA with variance partitioning based on the models' sum of squares, followed by Tukey's HSD post hoc tests. P-values were adjusted for multiple testing using the Benjamini–Hochberg FDR procedure. Full model outputs are provided in Supplementary Data 12.

Results Section

- The authors highlight 4 categories of functional enrichment terms in the results in Fig 3. Please provide 1-2 sentences in which you describe why these 4 were chosen specifically.

We thank the reviewer for requesting this clarification. We have added a brief rationale to the Results (Lines 211–216). As noted in the manuscript, displaying all differentially active pathways at once can obscure biological patterns, so we highlighted a focal subset of significantly upregulated pathways by grouping them into four broad thematic categories. We specifically chose these categories (carbon and nitrogen metabolism, vitamin biosynthesis, and bacterial interaction) because they capture the core metabolic and regulatory dynamics of the symbiosis.

- There are several statements in the results that are interpretive and are better suited for the discussion (e.g., lines 327-330).

We agree that the original phrasing (e.g., suggests, reinforcing) was overly interpretive for the Results section. We removed speculative language and moved the core spatial observation (the co-localization of NiR expression with photosynthetically fixed carbohydrates) into the hypothesis formulation that opens the next section (Lines 256–259). We also carefully reviewed the rest of the Results for similar phrasing and rephrased as needed.

Overall, I find this to be a rigorous and well-designed study with important implications for understanding holobiont nitrogen cycling. Pending addition of key methodological details, statistical transparency, and expanded discussion of limitations, it will make a valuable contribution suitable for publication in Nature Communications.

Reviewer #2 (Remarks to the Author):

Overall, this is a well-written and well-executed set of experiments. The data, I believe, could be explored further to yield a more impactful story and more actionable insights. I think it is worthy of publication, and my recommendation is minor revision. While I am recommending some data re-analysis, I do not think my suggestions go far beyond what the authors may have already done. I find it strange that there is no supplemental information included with this manuscript. Several of my suggested points may be better suited for a supplement, depending on their impact on the overall story. Nevertheless, I see these points as integral to presenting a comprehensive set of results for the reader.

What are the noteworthy results?

The paper describes a carefully controlled experiment with novel and informative results concerning the distribution of microbial species across cnidarian tissues in the presence and absence of algal symbionts. The results indicate shifts in the anemone's microbiome dependent on symbiotic state. The paper is unique in examining these trends in a tightly controlled and tissue-specific manner using laser microdissection.

In the introduction, the authors describe many microbiome studies as failing to explore functional implications in depth. While I appreciate the dual transcriptomic–metabolomic approach used here, I do not find the results to dig much deeper than other papers. KOG enrichments are standard for RNA-seq analysis, and although tissue-specific conditions are rare in cnidarian studies, the authors could extract more information and value from this dataset.

- Will the work be of significance to the field and related fields? How does it compare to the established literature? -

I find this work to be significant and to contribute to the field. A stronger and deeper understanding of cnidarian–algal symbiosis is essential for coral-bleaching mitigation and for investigating the evolution of endosymbiosis more generally. The paper adds important knowledge to the literature. In particular, it shows that:

1. nitrite assimilation pathways are favored in symbiosis;

2. certain essential amino acids are primarily produced by the symbionts, while levels of others are enhanced by particular microbes;

3. microbial communities in cnidarians exhibit tissue-specific compartmentalization, and these associations may be metabolically driven.

However, although I find the approach and methods impactful, the results presented are not, in my opinion, as impactful as they could be if the data were explored in greater detail. I offer some suggestions below that I think should be strongly considered for acceptance.

- Does the work support the conclusions and claims? Are there flaws in the data analysis or interpretation? -

The work does support the current conclusions. However, I am somewhat disappointed in the depth of analysis presented. This appears to be a potentially very rich dataset, yet the analyses are, in my opinion, somewhat surface-level. Below are several comments and suggestions for additional analyses or clarifications that could make this a more impactful contribution.

We thank the reviewer for the careful evaluation and specific suggestions for deeper analysis. We took the comment that the original submission did not fully exploit the dataset seriously, and have added substantial new analyses in response. In brief:

- We characterized the previously unlabeled pathway cluster in Fig. 3f and added complete GSVA scores and limma outputs as Supplementary Data 7 and 8.
- We audited the SymGas vs ApoGas differential expression distribution and clarified its biological interpretation in the Results.
- We added an integrated taxonomic re-analysis using MetaPro (mRNA community) and Kraken2 (rRNA community), with family- and genus-level breakdowns and alpha/beta diversity analyses (Supplementary Fig. 2, Supplementary Data 2–3).
- We processed and analyzed a previously held-back technical-replicate UHPLC-HR-MS injection; it shows strong concordance with the primary run (Pearson $r = 0.968$; Lin's CCC $= 0.964$) and recapitulates all biological patterns (Supplementary Fig. 5).
- We added molecular validation of the gnotobiotic state (16S rRNA PCR) as a new Fig. 4b, laser microdissection visuals as Supplementary Fig. 1, and per-sample sequencing quality metrics as Supplementary Data 1.
- We added a limitations paragraph to the Discussion and cross-referenced our previously published host-side spatial transcriptomic and metabolic data (Cui et al., 2023a, Science Advances) throughout the text, so the bacterial findings here are explicitly situated within the holobiont framework the reviewer emphasizes.

Our point-by-point responses follow.

1. Unannotated gene cluster in Figure 3F:

There is a cluster of genes at the top of the heat map that appears to have the strongest signal and interaction for gastrodermis and symbiont. There is no annotation of the potential functions in that cluster. Perhaps none were found, or perhaps the authors considered them irrelevant to nitrogen cycling, but they should be discussed. If no annotations were obtained, I suggest further BLAST searches against both microbial and eukaryotic databases; some sequences may be protist or anemone genes that slipped through filtering.

We thank the reviewer for this careful observation. In response, we revisited the cluster in detail.

First, we would like to clarify that the cluster is not unannotated: all transcripts contributing to it were processed through our standard KMAP/BLAST-based annotation pipeline, and pathway-level functional assignments are available for every member. The absence of labels in the original Fig. 3f was a presentation choice rather than an annotation gap. Our visualization focused on pathways significantly upregulated in symbiotic anemones (the active metabolic currencies underlying holobiont nutrient exchange), so we did not highlight this cluster, which shows the opposite trend (high GSVA scores in aposymbiotic samples that are progressively suppressed in symbiotic samples, with the strongest suppression in the symbiotic gastrodermis).

As is well known for KEGG-based functional annotation, a fraction of bacterial transcripts map to KEGG pathway diagrams originally created for eukaryotic or disease contexts (e.g., Chagas disease, RIG-I-like receptor signaling, Proteasome, Protein processing in endoplasmic reticulum). This occurs because these pathway maps include conserved enzyme families (heat shock proteins, ClpP-family proteases, conserved kinases) that are shared across all domains of life and are present in the bacterial transcripts under analysis. These mappings are therefore well-characterized artifacts of the KEGG pathway framework rather than evidence of host or algal contamination in the underlying microbial sequence assignments. We hope this addresses the reviewer's concern that some sequences might represent eukaryotic genes that slipped through filtering: the underlying transcripts are bacterial, but a subset of the pathway maps they map onto were originally drawn in eukaryotic contexts.

Setting these annotation artifacts aside, the cluster also includes several genuinely bacterial KEGG pathways with biologically coherent behavior, such as beta-lactam resistance (ko01501), the phosphotransferase system (ko02060), antifolate resistance (ko01523), epithelial cell signaling in Helicobacter pylori infection (ko05120), and cell motility (ko99993). Together, these represent antimicrobial defense, sugar uptake under nutrient stress, and motility/host-encounter functions. Their suppression in the symbiotic gastrodermis is biologically consistent with the bacterial community downregulating defense and free-living lifestyle programs once a stable symbiotic state is established, complementing the upregulation of metabolic exchange pathways shown in the lower portion of the figure.

We have updated Fig. 3f to label representative pathways from the top cluster, in addition to the previously annotated upregulated pathways. We have also added two new comprehensive supplementary tables: Supplementary Data 7 contains the raw GSVA scores for all pathways across all samples, and Supplementary Data 8 provides the full statistical results from the limma analysis. Readers can therefore inspect every pathway in the heatmap, not just only those highlighted in the figure.

2. Suspicious band in Figure 3E:

In the SymGas/ApoGas comparison, there is a strange band showing nearly no genes with ~2-fold change. That seems suspicious and suggests something may be off with the fold-change analysis for that comparison. The authors should investigate this further.

We thank the reviewer for the careful evaluation of Figure 3E. The observation prompted a thorough re-audit of the SymGas vs ApoGas differential expression. The reanalysis confirmed that the apparent depletion of genes around the ~2-fold change mark is not a computational artifact. We recalculated transcript-level fold changes directly from the normalized TPM matrix and found strong concordance with the sleuth effect-size estimates used in the figure (Pearson's r = 0.87), validating the underlying calculations.

Rather than an analytical error, this distribution reflects the high magnitude of biological restructuring in this specific tissue compartment. This contrast is dominated by large, SymGas-specific expression shifts: 63.2% of transcripts exhibited an |effect size| > 2, whereas only 5.0% fell near the log(2) interval. Furthermore, 84.7% of these large-effect genes arose from transcripts detected predominantly in the symbiotic gastrodermis but weakly or not at all in the aposymbiotic state. This distribution is consistent with the broader model presented in the manuscript. The symbiotic gastrodermis acts as a highly active hotspot for microbially mediated carbon and nitrogen metabolism. Because this compartment is directly exposed to photosynthetically derived carbon, the introduction of the algal symbiont triggers broad, high-magnitude activation of microbial functions (acting almost as an "on/off" switch) rather than moderate (~2-fold) regulatory tuning. We have revised the text (Lines 203–208) to provide a clearer interpretation.

3. Lack of anemone transcriptomic discussion:

The absence of analysis of anemone transcripts is surprising. Perhaps this is intended for another paper, but presenting the story holistically would be far more impactful. At minimum, I would like to know whether any complementary pathways are differentially regulated in the anemones—e.g., vitamin biosynthesis or metabolic pathways that might interact with changes in microbes or symbionts.

We agree with the reviewer that integrating the host's metabolic regulatory response is essential for understanding the complete symbiotic ecosystem. However, rather than being omitted from the broader narrative, this manuscript was specifically designed as the targeted bacterial complement to our recently published host-side spatial transcriptomic and metabolic analyses (Cui et al., 2023a).

In that paper, we characterized the anemone's complementary metabolic pathways and demonstrated how symbiosis induces coordinated upregulation and spatial relocalization of host glucose and ammonium transporters, which drives organism-wide nitrogen assimilation via the host GS-GOGAT cycle. The current study addresses the missing bacterial half of that metabolic equation. Using laser microdissection, we mapped how the bacterial microbiome dynamically responds to the specific carbon- and nitrogen-rich gastrodermal environment established by the host in Cui et al. (2023a). For instance, the massive upregulation of bacterial nitrite reductase (NiR) and subsequent microbial synthesis of essential amino acids reported here align perfectly with the host's spatial carbon provision and nitrogen assimilation pathways.

Because the host transcriptomic and metabolomic data are already published and publicly available, re-analyzing that host data falls outside the scope of this manuscript, which aims to unmask the novel functional contributions of the bacterial microbiome. We have, however, ensured that the functional links between the host's established metabolic state and the bacterial response are cross-referenced throughout the text, so readers can place the bacterial findings within the holistic framework the reviewer highlights.

4. Limited taxonomic resolution:

The text mentions B. subtilis and Klebsiella, but there is no broader taxonomic breakdown. Why is the data presented only at a high taxonomic level? If read quality or abundance prevents more precise taxonomic assignment, this should be clearly stated rather than left implicit.

We thank the reviewer for highlighting the need for broader taxonomic context. In response, we performed an integrated taxonomic re-analysis combining MetaPro (for the mRNA-level active community) and Kraken2 (for the rRNA-level standing community), and have added new supplementary resources covering family- and genus-level breakdowns, alpha and beta diversity, and an explicit discussion of taxonomic-resolution limits.

On the broader question of why the original manuscript focused on higher taxonomic ranks: in host-dominated metatranscriptomic datasets, fine-scale taxonomic assignment for the rare microbial biosphere is constrained by read quality, microbial biomass, and reference database coverage. Therefore, we structured the manuscript around functional metabolic shifts (pathway-level activity), which are more robust to these technical constraints than species-level identities. The new taxonomic analyses support this approach, showing that both the rRNA and mRNA communities are significantly restructured by tissue type and symbiotic state, but the functional shifts remain the clearest biological signal. To provide the comprehensive taxonomic context requested, we have added several new supplementary resources:

- Supplementary Data 2: taxonomic breakdowns at the family and genus levels for both the active (MetaPro) and standing (Kraken2) communities.
- Supplementary Data 3 and Supplementary Fig. 2: alpha and beta diversity analyses for both communities.
- Discussion (Lines 401–405): a limitations section outlining why fine-scale taxonomic assignments should be interpreted cautiously in host-dominated systems.

5. Lack of RNA data and microbial confirmation in gnotobiotic treatments:

Was there really no RNA-seq data for the gnotobiotic treatments? This would have enabled fascinating comparisons to separate symbiosis-specific changes from microbial influences. I would also have liked to see 16S abundance data from the gnotobiotic samples to confirm axenity. A single low-resolution photo of petri dishes is not convincing.

We agree that parallel metatranscriptomics on the gnotobiotic treatments would have produced a valuable comparative dataset. However, this was not technically feasible, as the antibiotic treatment depleted microbial biomass below the threshold required for reliable RNA-seq library preparation. This limitation is now noted in the Discussion (Lines 392–401) as an open technical challenge for the field. The ^{15}N stable isotope experiment was designed in part to compensate by providing direct functional validation of microbial metabolic contributions.

We also agree that plating alone is insufficient to confirm axenity. Alongside plating, we performed 16S rRNA gene PCR amplification (primers 67F/1492R; Lines 595–598) but inadvertently omitted this step from the original submission. We have now added the agarose gel image as Fig. 4b. The absence of a detectable 1.5 kb amplicon in gnotobiotic samples, combined with the plating results (Fig. 4a; Supplementary Fig. 4), provides evidence of microbial depletion in gnotobiotic samples.

6. Lack of visuals for laser microdissection:

I am surprised that there are no visuals of the laser microdissection process. Some should be included in the supplement.

We agree. We have expanded Supplementary Fig. 1 to include representative images of the laser microdissection process, showing the targeted cutting and capture of gastrodermal and epidermal tissue layers.

7. Insufficient statistics on sequencing quality:

I would also recommend including metrics on read quality, read depth, etc., by sample. as these are important in understanding the limitations of the conclusions that can be drawn from the data.

We agree. Per-sample sequencing QC, including raw read counts, post-filtering depth, and partitioning into rRNA and mRNA fractions, are now provided as Supplementary Data 1.

- Is the methodology sound? Is there enough detail for reproducibility? -

The methods and execution of the experiment are largely sound. However, a few points are unclear and would benefit from clarification for reproducibility and for interpretive context:

1. Brine shrimp sterility:

Were the brine shrimp also grown and hatched in sterile seawater? If so, how often and by what methods was the hatchery disinfected or sterilized between cultures?

We thank the reviewer for raising this critical point. Feeding is indeed a potential source for microbial contamination. To avoid this exact confounding variable, we did not feed the anemones during the microbe-depletion phase. All feeding was stopped at the start of the antibiotic treatment. The gnotobiotic anemones were maintained without food to ensure no exogenous microbes or organic matter were inadvertently introduced. This is now clarified in the Methods (Lines 589–590).

2. Feeding of gnotobiotic anemones:

Were the gnotobiotic anemones fed? This is important for reproducibility and for interpreting the metabolomics data. If they were not fed during/after antibiotic exposure, when were they last fed, and was this consistent across replicates and conditions?

The gnotobiotic anemones were not fed during the antibiotic treatment or the ^{15}N-tracer incubation. All anemones received their final feeding ~24 hours before the antibiotic protocol began, consistent across all biological replicates and conditions. This is now specified in the Methods (Lines 610–613).

3. Technical replicates in metabolomics:

Were any technical replicates performed for the metabolite analysis? From my reading, it seems not. If samples are still preserved, I suggest re-running with at least two technical replicates.

We appreciate the reviewer highlighting the importance of assessing technical variance in metabolomic analyses. Unfortunately, no preserved material is available for *de novo* re-runs. However, anticipating the need to safeguard against potential instrument variability, we performed dual injections (one primary run and one backup technical replicate) of all original sample extracts on the same analytical day during the initial UHPLC-HR-MS experiment. The original manuscript used only the primary injection because its spectral quality was high. Prompted by the reviewer's suggestion, we have now fully processed and analyzed this paired technical replicate as well.

Pearson correlation analysis across 192 paired measurements (4 experimental conditions × 8 essential amino acids × 2 treatments × 3 biological replicates) yielded a correlation coefficient of $r = 0.968$ ($R^2 = 0.937$, $p < 2.2 \times 10^{-16}$), and Lin's concordance correlation coefficient (CCC = 0.964) indicated strong agreement between the two injections with negligible systematic bias (Supplementary Fig. 5a). Furthermore, independent statistical analysis of the technical replicate data recapitulated the same biological patterns and group-level differences observed in the primary run (Supplementary Fig. 5b, c), showing that the conclusions are robust to technical instrument variance. The correlation analysis and the replicate-based enrichment analysis are provided as Supplementary Fig. 5. The dual-injection strategy is described in the Methods (Lines 652–659).

Reviewer #3 (Remarks to the Author):

The manuscript addresses an important and timely question in cnidarian holobiont biology and provides evidence that symbiogenesis with photosynthetic dinoflagellates drives a tissue-specific reorganisation of Aiptasia microbiome, with functional consequences for nitrogen assimilation at the holobiont level. These findings advance the field by linking spatial microbiome structure to holobiont metabolic function. The integration of tissue-resolved metatranscriptomics, gnotobiology and δ15N analyses is innovative and the study has the potential to make a significant impact. The data generally support the conclusions, and the methodology is sound; however, the manuscript currently lacks sufficient detail for reproducibility regarding statistical and isotopic analyses, and several figures require clarification. Addressing these issues will strengthen the manuscript and ensure it meets the standards expected for Nature Communications.

We thank the reviewer for the careful evaluation and the detailed, panel-by-panel suggestions. These prompted substantial improvments to the statistical reporting, figures, and supplementary materials.

We have taken the reviewer's two overarching concerns, the need for a dedicated statistical analysis section and the absence of supplementary information, as the structural backbone of this revision. We have added a dedicated Statistical Analysis section at the end of the Methods, consolidating model structures, post hoc procedures, and multiple-testing corrections in one place, in line with Nature Communications guidelines. Every quantitative claim in the Results now cites the test used and the adjusted p-value, with full model outputs (degrees of freedom, F-statistics, exact p-values, variance partitioning) provided in new supplementary materials. Particularly, we added:

- Per-sample sequencing quality metrics (Supplementary Data 1)
- Family- and genus-level taxonomic breakdowns of both the active (MetaPro) and standing (Kraken2) microbial communities (Supplementary Data 2)
- Alpha and beta diversity analyses (Supplementary Data 3; Supplementary Fig. 2)
- Global PCoA and PERMANOVA of the metatranscriptome across all 16 libraries (Supplementary Fig. 3)
- Technical-replicate validation of the UHPLC-HR-MS metabolomics data (Supplementary Fig. 5a; concordance r = 0.968, Lin's CCC = 0.964)
- Full GSVA scores and limma model outputs underlying Fig. 3f (Supplementary Data 7 and 8)
- Representative images of the laser microdissection process (Supplementary Fig. 1)

All figures have also been updated with explicit sample sizes, post hoc significance letters where appropriate, and clearer panel annotations as suggested.

Our point-by-point responses follow.

A dedicated and comprehensive Statistical Analysis section must be added to the Methods, following Nature Communications guidelines. Without this section, the robustness of the analyses cannot be evaluated. The manuscript appears not to include any Supplementary Information. If space becomes limiting when the authors add a proper statistical analysis section, some content could be moved to the Supplementary Materials.

We thank the reviewer for pointing out this structural requirement. In line with Nature Communications guidelines, we have consolidated all statistical details into a dedicated Statistical Analysis section at the end of our Methods, covering the testsused, significance thresholds, and multiple-testing corrections across all analyses.

We have expanded our supplementary materials (detailed in our overview above) to include sequencing QC metrics, expanded genus/family-level taxonomic breakdowns, active vs. standing community diversity metrics, technical replicate analyses for metabolomics, and representative visuals of the laser microdissection process. We have ensured these are clearly cross-referenced throughout the revised main text.

Figure 1:

Figure 1a: Archaea are absent from the bars. Please clarify whether they were not detected, filtered out, or omitted unintentionally.

Please report sample sizes for each group in boxplots.

We thank the reviewer for the comment. Archaea were indeed detected and included in our analysis, but they constitute <1% of total relative abundance across all samples and are visually indistinguishable from the baseline on a linear stacked-bar axis. We have clarified this in the Fig. 1a legend, noting that archaea are included but fall below visibility at <1%.

We have updated the Fig. 1 legend (and all subsequent boxplot figures) to report sample sizes explicitly (n = 4 biological replicates per group). We have also added compact letter displays to the boxplots to indicate significant pairwise differences (Tukey's HSD), with full ANOVA results and variance partitioning provided in Supplementary Data 9.

Figure 1b: The manuscript only describes most abundant bacterial groups, but should also discuss differences among the four tissue types compared that are not obvious from the boxplots. Please also comment on within-group variability.

We thank the reviewer for this helpful comment. We agree that phylum-level summaries (Fig. 1b) capture only the dominant shifts and do not resolve finer tissue-level differences. The less abundant phyla showed limited and inconsistent phylum-level variation, which is why the original main text interpretation focused on dominant groups. We have now added higher-resolution taxonomic analyses at the family and genus levels (Supplementary Fig. 2; Supplementary Data 2 and 3), which resolve tissue- and symbiosis-associated shifts not apparent at the phylum level.

Regarding within-group variability, the global PCoA on all 16 libraries (Supplementary Fig. 3) shows that between-group separation by tissue layer and symbiotic state is significant (two-way PERMANOVA, both $p = 0.0001$), and within-group dispersions are homogeneous (PERMDISP, all $p > 0.5$). This indicates that the observed separation reflects shifts in group centroids rather than differences in within-group spread, and this is now stated explicitly in the Results (Lines 189–193).

Although it is explained in the Results section, it remains rather confusing that the bacterial groups shown in Fig. 1b do not correspond to those represented in the boxplots. In the boxplot panels, it would be helpful to indicate clearly which of the groups displayed in Fig. 1b each boxplot refers to. In addition, I suggest using a different colour palette for the boxplots to avoid confusion with Fig. 1a and 1b. Post-hoc analyses after significant ANOVAs have to be performed and significant differences shown in figures. In cases where significant tissue × symbiosis interactions are detected, it would be helpful to elaborate on their biological relevance and interpret these effects more explicitly.

We thank the reviewer for these highly constructive suggestions, which have substantially improved Figure 1.

To bridge the visual gap between the phylum-level bar charts (Fig. 1b) and the order-level boxplots (Fig. 1c–e), we have updated the boxplot facet headers to explicitly state the parent phylum alongside the order name (e.g., Order Name [Phylum Name]).

For the color palettes, since the blue and orange scheme used in the boxplots represents a manuscript-wide convention for our symbiotic and aposymbiotic experimental groups, we instead applied a completely distinct color palette to the taxonomic bar charts in panels 1a and 1b. This modification uncouples the taxonomic identities from the experimental conditions without disrupting the visual consistency of the broader manuscript. We also ensured that the taxonomic color scheme remained consistent between Fig. 1b and Fig. 2d to enable direct comparison of phylum-level patterns across the rRNA- and mRNA-based community profiles.

Regarding statistical rigor, we conducted Tukey's HSD post hoc analyses for all significant ANOVAs and have now superimposed compact letter displays directly onto the boxplots to clearly denote significant pairwise differences.

Regarding the biological interpretation of tissue × symbiosis interactions, we now report the full interaction terms in Supplementary Data 9, which show that significant interactions correspond to tissue-specific microbial responses, for example, Actinomycetales and Bacillales are enriched specifically in the symbiotic gastrodermis (Lines 118–121). This tissue-specific response is central to the compartmentalized metabolic framework we propose.

The same comments apply to other figures, including sample sizes and missing post-hoc results.

Following the rigorous framework established for Figure 1, we have systematically audited all figures and corresponding statistical analyses throughout the manuscript.

Specifically, we have ensured that:

- Exact sample sizes (*n*) are explicitly stated in the figure legends for all boxplots and statistical charts across all figures.
- Post hoc significance testing (e.g., Tukey's HSD) has been conducted for all significant overarching tests (e.g., ANOVA).
- Compact letter displays denoting significant pairwise differences have been added to all relevant graphical panels.
- The full statistical outputs, including degrees of freedom, F-statistics, and exact *p*-values, have been compiled and added to the expanded Supplementary Data.

In line 270, you claim that bacteria and archaea are significantly enriched in the symbiotic gastrodermis (Fig. 2a,b); this requires supporting post hoc statistics. Viral abundances may not differ significantly between tissues. Please evaluate and report accordingly.

We appreciate the reviewer's careful evaluation of these claims. Following the comprehensive statistical audit implemented across our revised manuscript, we have fully addressed both points.

First, regarding the bacteria and archaea (Fig. 2a, b), we have conducted the required Tukey's HSD post hoc analyses after our two-way ANOVAs. The post hoc results statistically validate our original claim, confirming a massive and significant enrichment of both domains specifically within the symbiotic gastrodermis compared to all other tissue compartments ($p < 0.05$). To ensure complete transparency, these significant pairwise differences are now visually indicated using compact letter displays directly on the panels in the revised Figure 2, and the full statistical outputs are provided in Supplementary Data 10

Second, for viral abundance, our formal statistical evaluation confirmed that the main effect of Tissue alone does not significantly influence viral abundance (ANOVA, Tissue p = 0.098). However, we identified a highly significant main effect of Symbiosis (ANOVA, Symbiosis p = 0.0026). Consequently, post-hoc testing revealed that while viral abundance does not differ substantially across most compartments, there is a significant enrichment specifically within the symbiotic gastrodermis compared to aposymbiotic tissues. We have revised the main text in the Results section to accurately reflect these distinct statistical findings.

Figure 3:

The interpretation of microbial DEGs in line 296 can be further supported by group separation in the PCA plots. I suggest explicitly mentioning that PC1 explains >50% of the variance in Fig. 3b and 3d, compared to ~27% in 3a and 3c.

We thank the reviewer for this suggestion. We have updated the Results section (Lines 196–202) to incorporate these variance percentages (>50% vs. ~27%), using them as direct, quantitative support for the extensive DEG counts observed in the symbiotic gastrodermis.

Have the authors attempted a single PCA including all four groups simultaneously? If so, do the conclusions remain consistent?

Yes, we performed a global ordination including all four groups simultaneously, now shown in Supplementary Fig. 3, using PCoA on the sample-to-sample distance matrix that underpins PERMANOVA for statistical tests. This global ordination is consistent with the pairwise PCAs in Fig. 3a–d: symbiotic state and tissue layer both structure the microbial transcriptome, with symbiotic gastrodermis samples occupying the most distinct region of the ordination space. A two-way PERMANOVA on the same matrix confirmed significant effects of both symbiotic state (R^2 = 0.20, p = 0.0001) and tissue layer (R^2 = 0.23, p = 0.0001), with homogeneous within-group dispersions (PERMDISP, both $p > 0.5$). The conclusions of the manuscript are therefore unchanged.

Explanation of Fig. 3e in the figure caption requires improvement, including an explanation of why (only) expression of genes of nitrite reductase is shown. Which statistical test was performed?

We have revised the Fig. 3e legend to make clarify that the panel shows the genome-wide differential expression results from all four pairwise comparisons, with nitrite reductase (NiR)

transcripts highlighted in red rather than as the sole content. The biological rationale for focusing on NiR is explained in the main text following the figure (Lines 244–251), where we describe how GO enrichment analysis identified nitrate assimilation (GO:0042128) as one of the most over-represented biological processes in the SymGas comparisons, prompting us to examine the underlying NiR-coding transcripts. Thus, the NiR highlight is based on an unbiased GO-enrichment analysis rather than a pre-selected gene set. Statistical details (Wald test in sleuth, Benjamini–Hochberg adjustment) are now stated explicitly in the revised legend.

Lines 323–324 report p-values without specifying the test used. This must be corrected following the journal statistical-reporting guidelines. In general, I suggest the authors to improve statistical reporting in figures and within results according to the journal standards.

We thank the reviewer for catching this. The *p* values reported here are derived from Fisher's exact tests as implemented in topGO using the weight01 algorithm. We have revised the Results sentence to specify the test (Line 246), and expanded the Methods description (Line 563–572) of the GO enrichment analysis to state the algorithm, the underlying statistic, and the background gene set. We have also carried out a manuscript-wide review of statistical reporting in response to the reviewer's broader suggestion.

In Fig. 3f, please explain why some groups show high apparent microbial gene activity (intense red, on the top of the figure) but no specific pathways are indicated in those cases.

We thank the reviewer for this observation. The intense red signal at the top of Fig. 3f corresponds to a cluster of pathways with high GSVA scores in the aposymbiotic samples (ApoEpi and ApoGas) that are progressively suppressed in symbiotic samples, with the strongest suppression in the symbiotic gastrodermis. The absence of labels in the original figure was a deliberate a presentation choice: our visualization emphasized pathways significantly upregulated in symbiotic anemones, as these represent the active metabolic currencies underlying the holobiont nutrient exchange investigated in the manuscript.

Upon closer inspection of the cluster contents, we found it includes several biologically coherent bacterial pathways, such as beta-lactam resistance (ko01501), the phosphotransferase system (ko02060), antifolate resistance (ko01523), epithelial cell signaling in *Helicobacter pylori* infection (ko05120), and cell motility (ko99993). Together, these represent antimicrobial defense, sugar uptake under nutrient stress, and motility/host-encounter functions. Their suppression in the symbiotic gastrodermis is consistent with the bacterial community downregulating defense and

free-living lifestyle programs once a stable symbiotic state is established, complementing the upregulation of metabolic exchange pathways shown in the lower portion of the figure.

We have updated Fig. 3f to label representative pathways from this top cluster, in addition to the previously annotated upregulated pathways. We have also added two new comprehensive supplementary tables: Supplementary Data 7, which contains the raw GSVA scores for all pathways across all samples, and Supplementary Data 8, which contains the full statistical results from the *limma* two-way factorial analysis. These tables allow readers to inspect every pathway in the heatmap, not only those highlighted in the figure.

I am aware that compound-specific stable isotope analyses of amino acids are costly. Nevertheless, assessing the carbon sources of essential amino acids would have been valuable for more directly linking cnidarian nutrition with the contributions of microbial symbionts.

We agree with the reviewer that extending the analysis to amino acid carbon would directly address whether microbe-produced amino acids incorporate carbon from algal photosynthates. As the reviewer notes, however, such experiments are analytically demanding, and the initial design of this study, which focused on tracing nitrogen flow from inorganic substrates into amino acid pools, was established before the carbon-source question became apparent from our LC-MS data. We have added a sentence to the future directions paragraph of the Discussion (Lines 416–427) highlighting amino acid carbon tracing as a high priority next step for resolving the carbon aspect of the metabolic exchanges described in this work.

Figure 4:

In Figures 4b, 4c, and 4d, the values represent 15N enrichment relative to control samples. To clarify that the figure shows a difference from the control, rather than an absolute tissue value, it would be more accurate to label the axes as "15N enrichment" or "Δδ15N". It may also be worth noting in the results that in aposymbiotic organisms, several amino acids (Phe, Thr, Leu, Lys in Fig. 4b) display lower values relative to controls, an effect not observed for the amino acids shown in Fig. 4c.

We thank the reviewer for these helpful suggestions.

Regarding the axis label, we agree that $\Delta\delta^{15}N$ is more accurate than $\delta^{15}N$, since the plotted values represent the difference between ^{15}N-labeled treatment samples and unlabeled controls, rather than absolute isotopic composition. We have updated the y-axis labels of Fig. 4c–e, the figure legend, and all corresponding mentions in the Results to read $\Delta\delta^{15}N$ (‰).

Regarding the observation that several amino acids in Fig. 4c (Phe, Thr, Leu, Lys) show values at or below the unlabeled control baseline in aposymbiotic anemones, we interpret these values conservatively as indicating no detectable net ^{15}N incorporation above background under aposymbiotic conditions. The small negative deviations likely reflect background variability and/or dilution by pre-existing unlabeled amino acid pools during incubation. In contrast, the amino acids shown in Fig. 4d (Arg, His, Ile, and Trp) retained positive $\Delta\delta^{15}N$ values even in aposymbiotic anemones, consistent with an additional microbial contribution that is further enhanced under symbiotic conditions. We have added a sentence to the Results to make this contrast explicit (Lines 303–310).

For Figure 4, the same comments made for Figure 1 apply. Please indicate sample sizes clearly and report the results of the post-hoc tests.

We thank the reviewer for this comment. We have updated the Fig. 4 legend to clearly indicate sample sizes (n = 3 biologically independent samples per group for panels c–e), define all boxplot elements (center line = median, box bounds = interquartile range, individual points = biological replicates), and report the results of the post hoc tests (Tukey's HSD; significant groups indicated by letters above the boxes for panels c and d; pairwise unpaired two-sided *t*-tests for panel e). Full statistical procedures are now detailed in the Methods, with statistical outputs provided in Supplementary Data 12.
